# Inheritance of paternal DNA damage by histone-mediated repair restriction

**DOI:** 10.1038/s41586-022-05544-w

**Published:** 2022-12-21

**Authors:** Siyao Wang, David H. Meyer, Björn Schumacher

**Affiliations:** 1 Institute for Genome Stability in Aging and Disease, Medical Faculty, University Hospital and University of Cologne, Cologne, Germany; 2 Cologne Excellence Cluster for Cellular Stress Responses in Aging-Associated Diseases (CECAD), Center for Molecular Medicine Cologne (CMMC), University of Cologne, Cologne, Germany

**Keywords:** Genomic instability, Spermatogenesis, Double-strand DNA breaks, Epigenetics

## Abstract

How paternal exposure to ionizing radiation affects genetic inheritance and disease risk in the offspring has been a long-standing question in radiation biology. In humans, nearly 80% of transmitted mutations arise in the paternal germline^1^, but the transgenerational effects of ionizing radiation exposure has remained controversial and the mechanisms are unknown. Here we show that in sex-separated *Caenorhabditis elegans* strains, paternal, but not maternal, exposure to ionizing radiation leads to transgenerational embryonic lethality. The offspring of irradiated males displayed various genome instability phenotypes, including DNA fragmentation, chromosomal rearrangement and aneuploidy. Paternal DNA double strand breaks were repaired by maternally provided error-prone polymerase theta-mediated end joining. Mechanistically, we show that depletion of an orthologue of human histone H1.0, HIS-24, or the heterochromatin protein HPL-1, could significantly reverse the transgenerational embryonic lethality. Removal of HIS-24 or HPL-1 reduced histone 3 lysine 9 dimethylation and enabled error-free homologous recombination repair in the germline of the F_1_ generation from ionizing radiation-treated P_0_ males, consequently improving the viability of the F_2_ generation. This work establishes the mechanistic underpinnings of the heritable consequences of paternal radiation exposure on the health of offspring, which may lead to congenital disorders and cancer in humans.

## Abstract (web summary)

In *Caenorhabditis elegans*, paternal exposure to ionizing radiation results in HIS-24 and HPL-1-dependent genome instability phenotypes, causing embryonic lethality in the offspring.

## Main

Ionizing radiation induces DNA double strand breaks (DSBs) that can cause de novo mutations (DNMs) and chromosomal aberrations^2^. Somatic DNMs are highly correlated with cancer and aging^3,4^. Germline DNMs can be transmitted to offspring and are a driving force of genome evolution^5^, but may also result in congenital diseases^6^. In humans, nearly 80% of DNMs^1^—including 70% of de novo structural variants^7,8^—originate from the paternal germline. Increasing evidence suggests that the paternal germline DNMs are associated with various congenital disorders, schizophrenia, autism and reproduction defects^1,9,10^. The correlation between parental exposure to ionizing radiation and genetic effects in the progeny has nonetheless remained uncertain^11–13^. Contradictory observations have been obtained from children born in the vicinity of nuclear power plants^14–16^. Studies on the progeny of the clean-up workers from the accident at Chernobyl and of survivors of the atomic bombings at Hiroshima and Nagasaki showed no evidence of a transgenerational effect of parental radiation exposure^17–20^. However, epidemiologic studies in humans are typically limited by the sample size, the mixed population, and records of radiation doses. By contrast, paternal exposure to chemotherapy before conception has recently been correlated with germline hypermutation^21^, which is associated with rare genetic diseases in the progeny^22^. This raises the question of whether specifically paternal exposure to mutagenic agents could lead to a transgenerational effect.

Here, we used sex-separated mutants of *C. elegans* to investigate the transgenerational effects of paternal radiation exposure. We show that paternal exposure to ionizing radiation results in genome instability in the F_1_ generation and transgenerational embryonic lethality. We determined that the paternal DNA damage is mainly repaired in the zygote through maternally provided error-prone polymerase theta-mediated end joining (TMEJ), which results in chromosomal aberrations. Structural variants with such TMEJ signature microhomology are also present in natural *C. elegans* variants and in paternal germline mutations in humans, suggesting a conserved mechanism. Removing the histone H1 HIS-24 or the heterochromatin protein HPL-1 could reverse the transgenerational embryonic lethality of paternal exposure to ionizing radiation by activating the error-free homologous recombination repair (HRR) pathway. Our work provides the mechanistic underpinnings of the transgenerational genetic and epigenetic effect of paternal exposure to ionizing radiation.

## Ionizing radiation induces a transgenerational effect

**Fig. 1:**
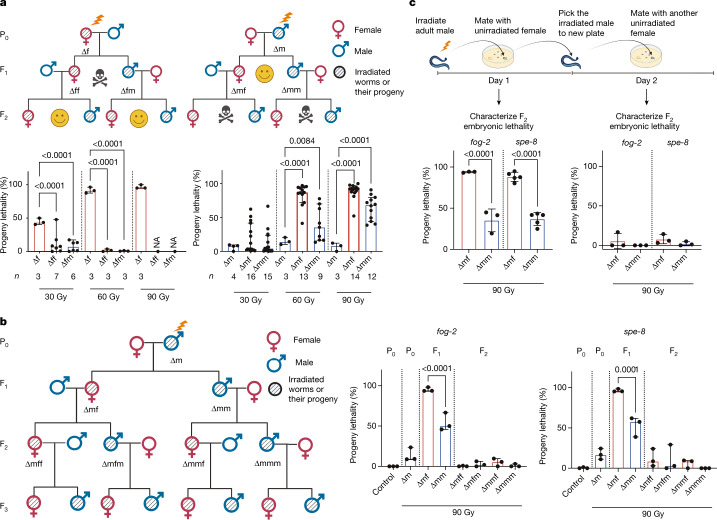
Paternal exposure to ionizing radiation leads to transgenerational embryonic lethality. **a**, Pedigrees of the maternal and paternal exposure to different doses of ionizing radiation and transgenerational characterization. Left, maternal exposure to ionizing radiation (P_0_ ∆f) leads to intergenerational embryonic lethality in the F_1_ generation. Except for the group treated with 90 Gy, which has no surviving progeny developing to adulthood, the surviving *fog-2* F_1_ (∆ff and ∆fm) crossed with non-irradiated opposite sexes recovered the embryonic lethality to basal level. *n* indicates the number of biological replicates; each replicate includes three *fog-2* females and three *fog-2* males. Right, paternal ionizing radiation exposure (P_0_ ∆m) leads to a mild increase in embryonic lethality in the F_1_ generation, and the progeny of the surviving F_1_ (∆mf and ∆mm) show a transgenerational embryonic lethality in the F_2_ generation. *n* indicates the number of biological replicates. Data are median ± 95% confidence interval; *P* values are shown. Throughout the figures, red bars indicate females and blue bars indicate males. NA, not applicable. **b**, Pedigrees of paternal ionizing radiation exposure (90 Gy) and the transgenerational lethality characterization for three consequent generations. *n* = 3 biological replicates, each replicate includes 3 females with 3 males. Data are median ± 95% confidence interval; *P* values are shown. **c**, Freshly irradiated P_0_ adult males and males two days after ionizing radiation exposure show different transgenerational effects. *fog-2*: *n* = 3 biological replicates; *spe-8*: *n* = 5 biological replicates; each replicate includes 3 females and 3 males. Data are median ± 95% confidence interval; *P* values are shown. Generalized linear model (GLM) with logit link function and Tukey’s multiple comparisons were used for proportional data, and one-way (**a**,**b**) or two-way (**c**) ANOVA with arcsine transformation was also used to confirm the statistical results (Supplementary Table 1).

To investigate the transgenerational consequences of genotoxic stress, we exposed *C. elegans* to ionizing radiation and assessed the consequences in the subsequent generations. Wild-type *C. elegans* has two sex forms: the self-fertilizing hermaphrodites and males. To distinguish the effect of DNA damage in male and female gametes, weused the feminized mutant *fog-2*, which has a dioecious reproductive system: somatic hermaphrodites that only generate a female germline and males with a normal male germline. Immediately after ionizing radiation treatment, females (∆f) were mated with unirradiated males and the viability of the progeny was characterized 24 h later. As expected, ionizing radiation treatment led to a dose-dependent embryonic lethality (Fig. 1a; full statistical analyses for all results are shown in Supplementary Table 1), which is attributed to the excessive unrepaired DNA DSBs in oocytes. When we crossed the surviving female (∆ff) and male (∆fm) F_1_ worms with healthy worms of the opposite sex, the F_2_ generation showed nearly no embryonic lethality (Fig. 1a). This observation indicates that the DNA DSBs carried in the oocytes can either be properly repaired or eliminated, thereby preventing any transgenerational effect from the maternal DNA damage.

We next tested whether ionizing radiation treatment in males (∆m) could affect subsequent generations. Immediately after ionizing radiation exposure, males were mated with untreated females. The progeny lethality of ∆m was relatively low (Fig. 1a), indicating that the paternal DNA damage had either been repaired or escaped from the checkpoint surveillance during the embryogenesis of the F_1_ generation. Unexpectedly, after crossing the F_1_ worms with healthy worms of the opposite sex, the embryonic lethality of the F_2_ generation was markedly increased (Fig. 1a). Notably, this increase in embryonic lethality is significantly different between the progeny of female F_1_ (∆mf) and the progeny of male F_1_ (∆mm) worms (Fig. 1a). These observations establish that male exposure to ionizing radiation can lead to transgenerational lethality in the F_2_ generation.

Subsequent mating of the surviving progeny from the F_2_ generation with the healthy opposite sex restored the viability of the progeny (Fig. 1b), indicating that the sperm-inherited detrimental effect can be largely eliminated within two generations of intercrossing with healthy worms. We also verified this observation with an independent feminized mutant, *spe-8* (Fig. 1b).

Probably owing to the limited DNA repair capacity within a highly condensed chromosomal architecture, mature sperm is more vulnerable than spermatocytes to DNA damage^23^. As in humans, spermatogenesis in male *C. elegans* is continuously ongoing in adults, and in one nematode mating about two-thirds of the mature sperm are ejaculated. To test whether the mature sperm might be responsible for the transgenerational lethality, we first collected the progeny derived from freshly irradiated *fog-2* or *spe-8* males to assess the effect of irradiation on mature sperm. On the second day, we mated those irradiated males with untreated females to test the effect of irradiation on spermatogenesis before maturation. Only the progeny derived from the freshly irradiated males led to transgenerational lethality (Fig. 1c), indicating that only mature spermatozoa were responsible for the ionizing radiation-induced transgenerational lethality.

**Extended Data Fig. 1:**
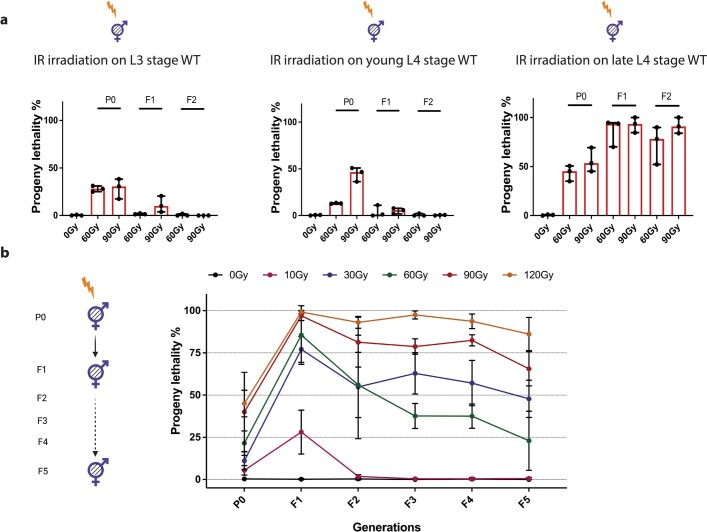
IR exposure of hermaphrodite WT in the late L4 stage leads to extended transgenerational embryonic lethality. **a**. Transgenerational lethality characterization for IR treated WT hermaphrodites at the L3 (start of spermatogenesis), young L4 (ongoing spermatogenesis) and late L4 stage (mature sperm is stored in the spermatheca). Ten worms were included for each biological replicate, and n = 3 biological replicates. Error bars indicate median with 95% CI. **b**. Progeny lethality characterization for multiple generations upon IR treatment at the late L4 stage of hermaphrodites P0. Ten worms for each dosage group were included for each biological replicate, and n = 3 biological replicates. Error bars indicate median with 95% CI.

We next tested whether the transgenerational lethality also occurs in wild-type *C. elegans* hermaphrodites. Spermatogenesis is initiated at the late L3 larval stage and ceases by the late L4 larval stage, where the germline switches to oogenesis while the mature sperm is stored in the spermatheca. Only irradiation of wild-type hermaphrodites at the late L4 stage but not at late L3 or young L4 led to transgenerational lethality in the F_2_ and F_3_ generations (Extended Data Fig. 1a), validating that ionizing radiation exposure of mature sperm in hermaphrodite wild-type worms could lead to transgenerational lethality.

In contrast to *fog-2* mutants, wild-type hermaphrodites self-fertilize and might thus further perpetuate the unstable genomes. Indeed, we observed that the transgenerational lethality was maintained over at least six generations (Extended Data Fig. 1b). These results suggest that the recovery of viability in the F_3_ generation of *fog-2* mutants is mainly owing to the dilution of the transgenerational effect by outcrossing the damaged genomes three times with the unirradiated opposite sex.

## Paternally induced genome instability

**Fig. 2:**
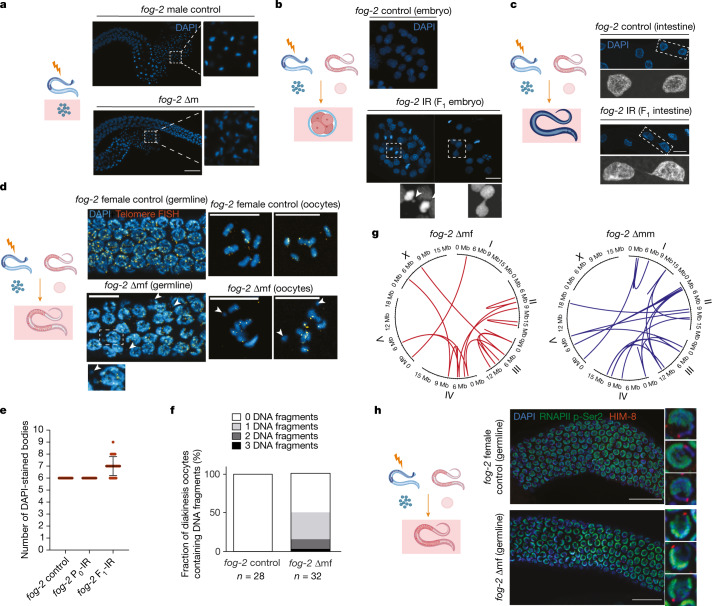
Paternal exposure to ionizing radiation leads to DNA fragmentation, chromosomal rearrangement and aneuploidy in the F_1_ generation. **a**, Left, male germline DAPI staining. Right, magnified mature sperm. Scale bar, 20 μm. **b**, Embryonic DAPI staining. Bottom, chromosomal lagging and bridging. Scale bar, 10 μm. **c**, Intestinal DAPI staining. Bottom, magnified view of two representative cells. Scale bar, 10 μm. **d**, Telomere fluorescence in situ hybridization (FISH) and DAPI staining in the germline. Bottom, magnified view of fragmented DNA. Right, late diakinesis oocytes. Arrowheads indicate DNA fragmentation. Scale bars, 10 μm. Experiments in **a**–**c** were repeated three times with similar observations; experiment in **d** was repeated two times with similar observations. **e**, Quantification of DAPI-stained bodies in late diakinesis oocytes. Data are mean ± s.d., *n* = 25 oocytes. **f**, Quantification of DNA fragmentation (without telomere FISH signal) per late diakinesis oocyte. *n* represents the number of oocytes. **g**, Representative circos plots showing chromosomal translocations (inter- or intra-chromosomal fusions) in ∆mf and ∆mm. All translocations are listed in Supplementary Table 3. **h**, RNAPll p-Ser2, HIM-8 and DAPI staining in dissected germline. Right, three representative nuclei. Scale bar, 20 μm. This experiment was repeated three times with similar observations. IR indicates exposure to ionizing radiation. Drawings illustrate the (**a**) sperm, (**b**) F_1_ embryos, (**c**) F_1_ somatic tissues and (**d**, **h**) F_1_ germlines of irradiated males that are investigated in the respective panels.

**Extended Data Fig. 2:**
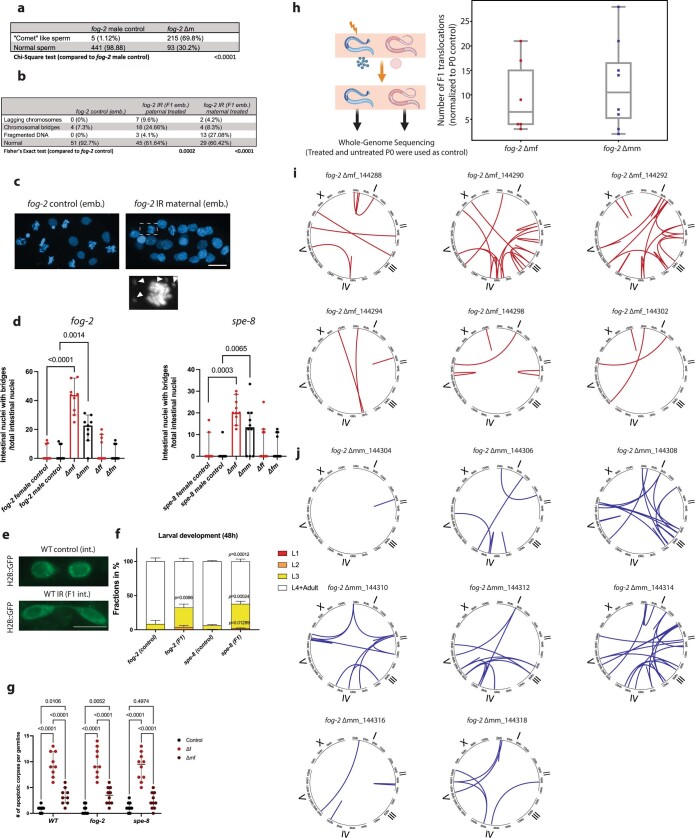
Paternal exposure to IR leads to genome instability in the F1 generation. **a**. Quantification of “comet” like sperm (p-values and n number indicating sperm are shown, Chi-square test was performed). **b**. Quantification of the chromosomal aberrations in the F1 of paternal and maternal exposure to IR (p-values and n number indicating embryos are shown, Fisher’s exact test was performed). **c**. Representative fluorescence microscopic images of DAPI (blue) in the *fog-2* embryos derived from the control or irradiated females. Zoomed image of chromosomal fragmentation is shown below, fragmented DNA is indicated with arrowheads. Scale bar = 10 μm. **d**. Quantification of the intestinal chromosomal bridges in the F1 progenies of irradiated males and females (n *fog-2* = 9, n *spe-8* = 10 intestines, medians with 95% CI are shown, p-values are shown, two-factor GLM with logit link function and Tukey multiple comparisons were used). Two-way ANOVA with arcsine transformation was also used for the proportion data to confirm the statistical results and included in the full statistic results shown in Supplementary Table 1. **e**. Representative fluorescence microscopic images of hermaphrodite WTs’ intestinal cells expressing H2B::GFP with or without parental IR irradiation (90 Gy). Chromosomal bridging was observed in the F1’s intestinal cells. Scale bar = 10 μm. **c** and **e** were repeated three times with similar results. **f**. Developmental stages were determined 48h post L1 stage of control group of *fog-2* and *spe-8* and the F1 generation of *fog-2* and *spe-8* with paternal IR exposure, and n = 30 individuals were analyzed per experiment (n = 3 independent experiments per strain, p-values are shown, two-tailed t-test was performed). Full statistic results are shown in Supplementary Table 1. **g**. Quantification of apoptotic corpses per germline of unirradiated control, 90Gy treated ∆f and ∆mf of WT, *fog-2* and *spe-8* mutants (n = 9 germline, Medians with 95% CI are shown, p-values are shown, Two-way ANOVA with Bonferroni’s multiple comparisons test were used). Full statistic results are shown in Supplementary Table 1. **h**. Number of new chromosomal translocations in ∆mf (n = 6 worms) and ∆mm (n = 8 worms). The box depicts the Median with the top and bottom quartiles, the whiskers extend to 1.5 IQR. New translocations and mapped reads per sample are shown in Supplementary Table 2. **i**. and **j**. Circos plots showing the novel chromosomal translocations in six *fog-2* ∆mf (**i**) and eight *fog-2* ∆mm (**j**). The list of all translocations is shown in Supplementary Table 3.

To address whether the paternal DNA damage causes heritable changes, we tracked chromatin states from the irradiated sperm to the germ cells of the F_1_ generation. Ionizing radiation treatment led to morphological changes in the sperm of *fog-2* ∆m with a ‘comet-like’ conformation (Fig. 2a and Extended Data Fig. 2a). After fertilization, the embryos showed various chromosomal instability phenotypes, including the formation of chromosomal bridges and chromosome lagging (Fig. 2b and Extended Data Fig. 2b). The formation of chromosomal bridges indicated that the dividing embryonic cells entered a ‘breakage–fusion–bridge’ cycle^24^. Despite these chromosomal aberrations, the majority of embryos developed into phenotypically healthy adults, suggesting that the DSBs in the sperm bypass the surveillance of mitotic checkpoints during early embryogenesis. Unlike the paternally irradiated embryos, DNA damage induced by maternal exposure to radiation mainly caused fragmented DNA in the F_1_ embryos (Extended Data Fig. 2b,c). Such DNA fragmentation could further trigger mitotic catastrophe, accounting for the high embryonic lethality in the F_1_ generation.

We next characterized the chromosomal states in the adult *fog-2* F_1_ offspring with paternally inherited DNA damage. Intestinal nuclei that undergo a set of programmed endoreduplications during larval development displayed chromosomal bridges in the *fog-2* F_1_ offspring of *fog-2* ∆m (Fig. 2c and Extended Data Fig. 2d), indicating the persistence of DNA breaks from embryogenesis until adulthood. We observed similar chromosomal bridges in the hermaphrodite F_1_ offspring from irradiated L4 larvae (Extended Data Fig. 2e). The intestinal chromosomal bridges were absent in the F_1_ generation of maternal radiation exposure, indicating that the genome stability of surviving F_1_ progeny from irradiated mothers was restored (Extended Data Fig. 2d). This observation was also confirmed in *spe-8* mutants (Extended Data Fig. 2d).

We also observed a developmental growth delay in the ∆mf and ∆mm of both *fog-2* and *spe-8* mutants (Extended Data Fig. 2f), suggesting that paternal exposure to ionizing radiation can lead to phenotypic consequences in the progeny.

In the pachytene zone of the germline of *fog-2* ∆mf worms, we observed DNA fragments (Fig. 2d, left) that were devoid of telomere sequence (Fig. 2d, left). Instead of the normal six highly condensed and discrete oocyte bivalents, meiotic diakinesis cells of the *fog-2* ∆mf germlines contained a larger number of DAPI-stained bodies that were irregularly sized, with some carrying randomly allocated telomere sequences (Fig. 2d, right, e,f). The chromosomal rearrangement, fragmentation and aneuploidy in the F_1_ generation might also provide an explanation for why the transgenerational effect is limited to three generations in the *fog-2* mutants but lasts more than six generations in hermaphrodite wild-type worms, as out-crossing with normal haploid gametes could greatly rescue the inheritance of aneuploidy.

The chromosomal aberrations prompted us to test whether the apoptotic DNA damage checkpoint was activated in the F_1_ germline. As the male germline lacks this apoptotic response^25^, we quantified the apoptotic corpses in the germlines of control, ∆f and ∆mf worms. Ionizing radiation induced a significant increase of apoptosis in the germlines of ∆f worms; however, only a mild increase of apoptosis was detected in the germline of ∆mf (Extended Data Fig. 2g) indicating that the paternally inherited genome instability does not trigger the DNA damage checkpoint in the F_1_ germline.

To detect de novo genomic aberrations in the *fog-2* F_1_ generation, we performed whole-genome sequencing (WGS) of single P_0_ and their direct F_1_ progeny. We detected between 3 and 21 de novo translocations (inter- or intra-chromosomal fusions) in each female (∆mf) and between 2 and 28 de novo translocations in each male (∆mm) F_1_ individual. In total, we mapped 154 translocations in the genomes of *fog-2* ∆mf and *fog-2* ∆mm worms compared with the parental generation (Extended Data Fig. 2h and Supplementary Table 2), whereas tandem duplications (*n* = 1) and insertions (*n* = 1) were rare (Supplementary Table 3). All de novo translocations in the F_1_ worms were heterozygous, consistent with only paternally inherited genomic aberrations. The genomes of female and male progeny showed an overall similar number of autosomal translocations. The paternal X chromosome is inherited in *fog-2* ∆mf but not in *fog-2* ∆mm worms, which carry only a maternal X chromosome. Consistently, only the *fog-2* ∆mf worms bear X chromosomal translocations (Fig. 2g, Extended Data Fig. 2i,j and Supplementary Table 3), indicating that the translocations occur only in the paternal chromosomes.

**Extended Data Fig. 3:**
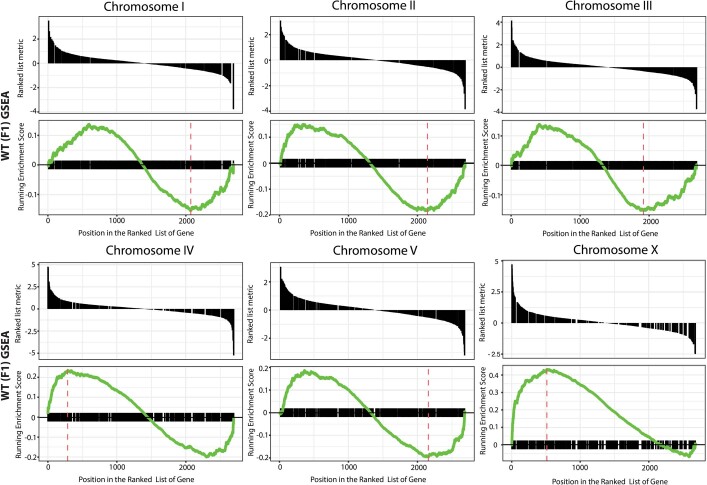
Gene set enrichment analysis on Chromosome I to Chromosome V of SILAC proteomic data from hermaphrodites WT (F1) with parental IR exposure. Gene set enrichment analysis (GSEA) of SILAC proteomic data from hermaphrodites WT (F1) with parental IR exposure (90 Gy) for chromosomes I to V. The top of each panel shows the log2 fold changes of the proteomics data in a ranked order. The bottom panels show the running enrichment score as a green line. The dashed red line depicts the positions furthest from 0. None of the chromosomes, except the X chromosome, is significantly different from a random distribution. The full statistics for each chromosome is provided in the Supplementary Table 4.

The X chromosomal translocations might be responsible for the particularly high progeny lethality in the *fog-2* ∆mf, as the integrity of the X chromosome is essential for meiotic sex chromosomal inactivation (MSCI). When the continuity of the X chromosome is lost, X chromosomes cannot enter the MSCI transcriptional inactivation state, resulting in high progeny lethality^26^. To assess whether MSCI is disrupted, we stained a transcription active mark, Ser2 phosphorylation (p-Ser2) of RNA polymerase II (RNAPII). In the pachytene region of the germline, X chromosomes (labelled with HIM-8 antibody, marking the X chromosome pairing centre) in the untreated *fog-*2 females were devoid of RNAPII p-Ser2, whereas in the *fog-2* ∆mf, RNAPII p-Ser2 was evenly distributed on both autosomes and X chromosomes (Fig. 2h). Consistently, gene set enrichment analysis (GSEA) for chromosomal gene distributions indicates a strong upregulation of X chromosomal proteins in the F_1_ generation of irradiated wild-type worms (Extended Data Fig. 3 and Supplementary Table 4), whereas no other chromosome was significantly enriched (Extended Data Fig. 3 and Supplementary Table 4). These observations suggest that the X chromosome rearrangement in the germline of *fog-2* ∆mf disrupts the MSCI process, which together with chromosomal translocations and fragmentation on autosomes, results in significantly higher progeny lethality compared with the *fog-2* ∆mm.

**Extended Data Fig. 4:**
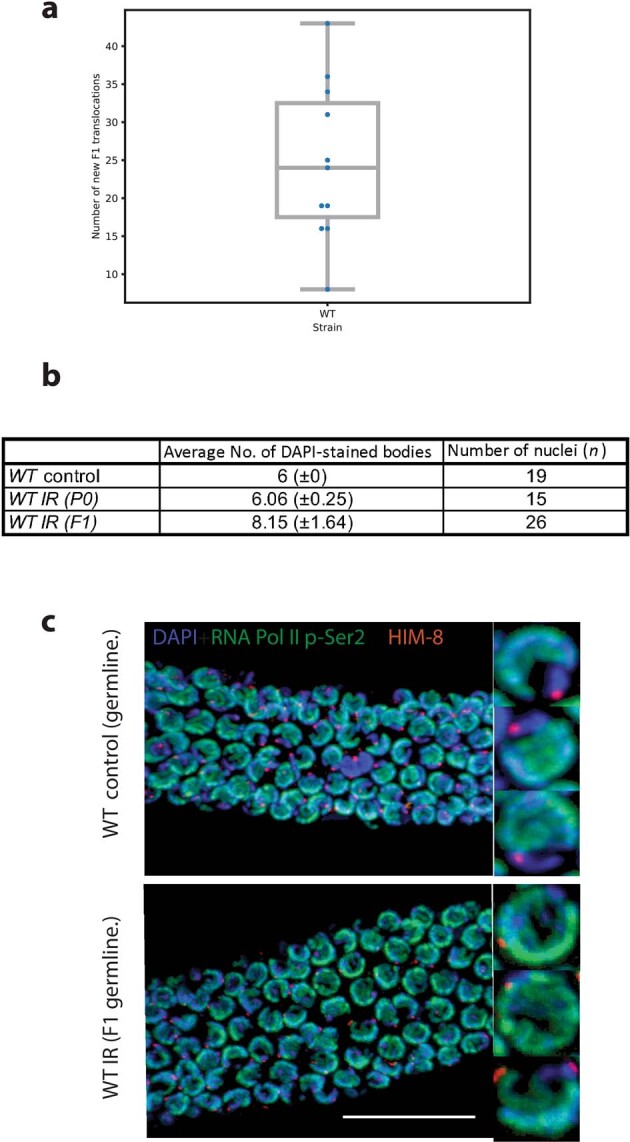
IR exposure in the late L4 stage of hermaphrodite WT leads to chromosomal rearrangement. **a**. Whole-genome sequencing analysis shows the number of new chromosomal translocations in the hermaphrodites WT adults (F1) with parental IR exposure (90 Gy), normalized to untreated control. n = 11 worms, the box depicts the Median with the top and bottom quartiles, while the whiskers extend to 1.5 IQR. The number of new translocations and of mapped reads per sample is shown in Supplementary Table 2. **b**. Characterization of the DAPI-stained bodies in the late diakinesis oocytes of hermaphrodites WT adults (F1) with or without parental IR exposure (90 Gy). **c**. Merged images of immunofluorescence co-staining RNA Pol ll p-Ser2 (green), HIM-8 (red) and DAPI (blue) of dissected germlines from hermaphrodite WT adult (F1) with or without parental IR exposure (90 Gy). Three representative nuclei are shown on the right side. Scale bar = 20 μm. This experiment was repeated three times with similar results.

**Extended Data Fig. 5:**
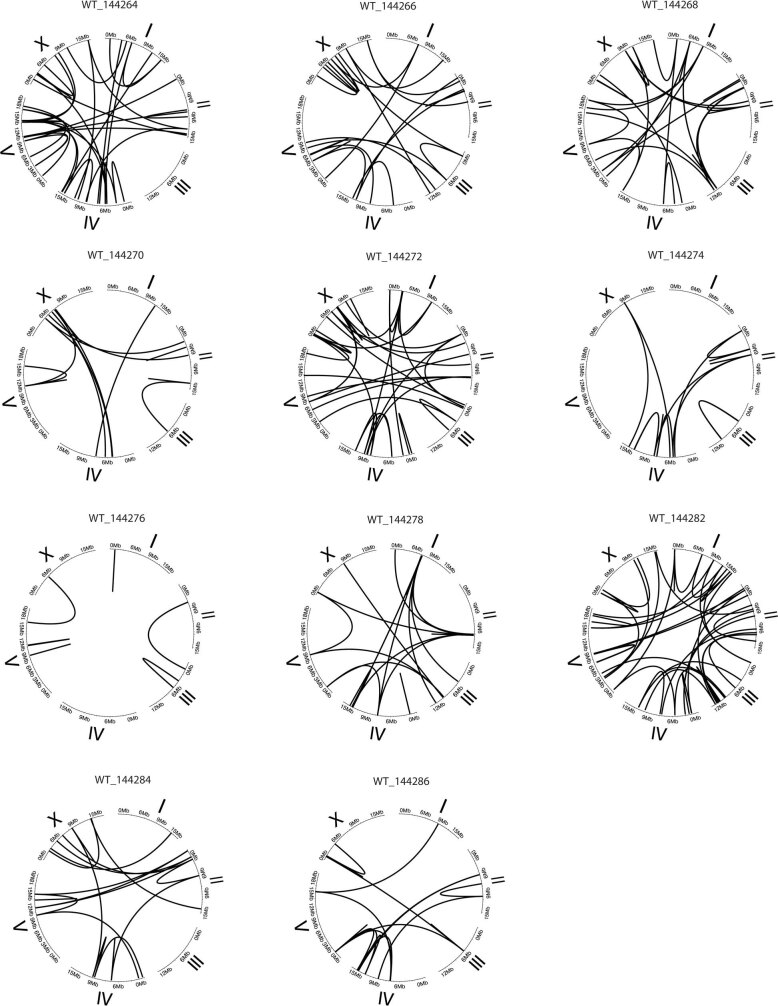
Circos plots showing the chromosomal translocations in ten hermaphrodites WT (F1) with parental IR (90 Gy) exposure. The list of all translocations is shown in Supplementary Table 3.

In addition to *fog-2* mutants, we also performed WGS of single hermaphrodite wild-type F_1_ worms and their P_0_ parents. Unlike the dioecious *fog-2* system, the self-fertilized hermaphrodites carried roughly twice the number of heterozygous translocations in the genome of their F_1_ generation (Extended Data Figs. 4a and  5). This could be explained by two possibilities: (1) the sperm of the hermaphrodites is more vulnerable than the sperm produced in the male worms; (2) in contrast to self-fertilizing wild-type hermaphrodites, mating of the *fog-2* males with healthy females providing a set of undamaged maternal chromosomes could greatly increase genome stability. Similarly to the above result, we also observed aneuploidy phenotypes and de-silencing of the X chromosome in the F_1_ of irradiated hermaphrodite wild-type worms (Extended Data Fig. 4b,c).

## Maternal TMEJ repairs paternal DNA

**Fig. 3:**
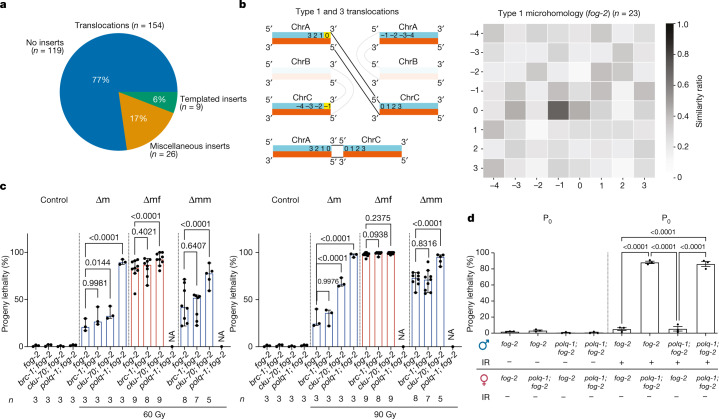
Paternally inherited DNA damage is mainly repaired by TMEJ. **a**, The distribution of translocation footprints in *fog-2* F_1_ adults with paternal exposure to 90 Gy ionizing radiation. Templated inserts are insertions of ≥3 bp in between the fusion sites that have a matching sequence within ±25 bp around one of the 2 break points. Miscellaneous insertions are insertions <3 bp or insertions with no matching sequence within ±25 bp around the breakpoints. *n*, number of translocations. **b**, Left, schematic illustration of the type 1 translocations. The sense strand is in blue, and the antisense strand is in red. Numbers indicate the distance of the nucleotide to the breakpoint; yellow boxes indicate positions that show microhomology. Right, heat map representing the sum of all type 1 translocation maps derived from ∆mf and ∆mm (*n* = 23 translocations), excluding translocations with a templated insertion. Darker shades indicate higher sequence similarity between the corresponding bases. Numbers along the *x*- and *y*-axes correspond to those on the left. *P* values are provided in Supplementary Table 1. **c**, Progeny lethality of indicated strains. *n* represents the number of biological replicates. Data are median ± 95% confidence interval. *P* values are shown. **d**, Progeny lethality of different parental combinations for *polq-1* worms with and without exposure to 90 Gy ionizing radiation. *n* = 3 biological replicates. Data are median ± 95% confidence interval. *P* values are shown. GLM with logit link function and Tukey multiple comparisons were used for proportional data, and one-way (**c**) or two-way (**d**) ANOVA with arcsine transformation was used to confirm the statistical results. Full statistical analyses are provided in Supplementary Table 1.

**Extended Data Fig. 6:**
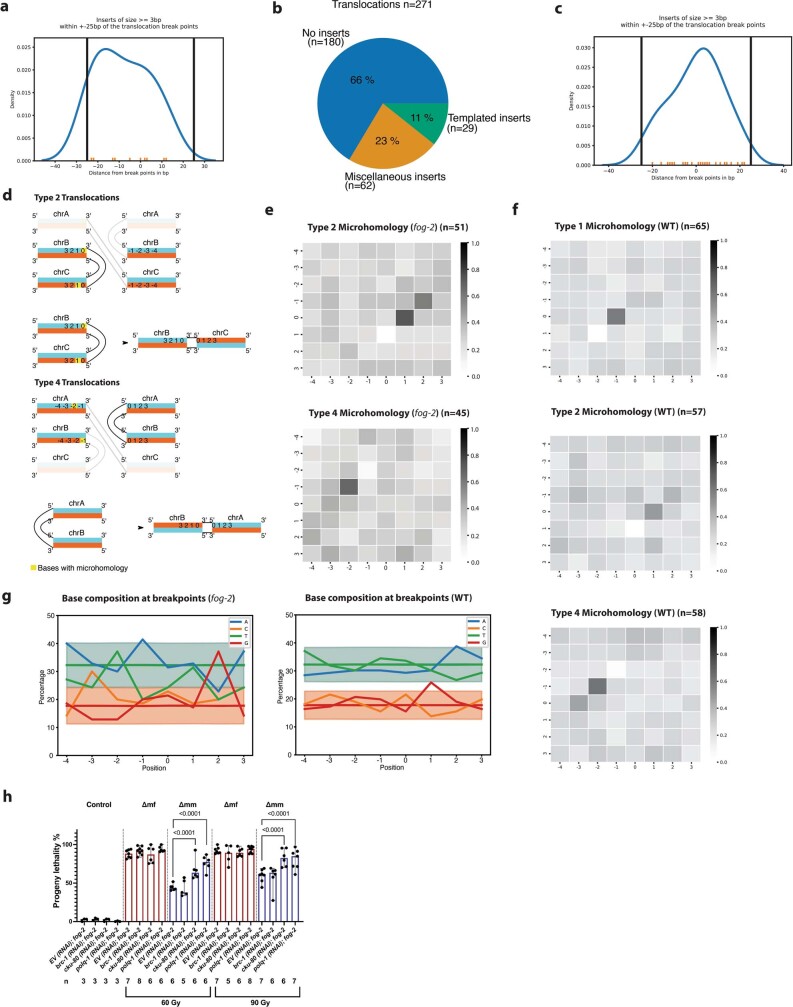
Hermaphrodite WT animals use TMEJ to repair the inherited DNA damage. **a**. Density plot showing the distribution of the template positions of the insertions found in between the translocation sites of *fog-2* adults (F1) with paternal exposure to IR (90 Gy). Only insertions are included that are at least 3 bp and whose templates were found within ±25 bp around one of the breakpoints (corresponding to the templated insertions in Fig. 3A). The orange ticks on the x-axis show the positions where the templates were found. The blue curve depicts the kernel density estimation as a non-parametric way to estimate the probability density function of the found positions. The black lines indicate the distance cutoff of ±25 bp from the breakpoints. **b**. Distribution of translocation footprints in the WT adults (F1) with paternal exposure to IR (90 Gy). Templated inserts are insertions of ≥ 3 bp in between the fusion sites that have a matching sequence within ±25 bp around one of the 2 breakpoints. Miscellaneous insertions are insertions < 3 bp or insertions with no matching sequence within ±25 bp around the breakpoints. **c**. The same density plot as in (A) but for WT adults (F1) with paternal exposure to IR (90 Gy) (corresponding to the templated insertions in Extended Data Fig. 3B). **d**. Schematic illustration of the type 2 (top) and type 4 (bottom) translocations. The sense strand is in blue, while the anti-sense strand is in red. The numbers indicate the nucleotide distance to the breakpoints. Type 2 translocations are fusions between the 3′ sense strand with the 5′ anti-sense strand. Type 4 translocations are fusions between the 3′ anti-sense strand with the 5′ sense strand. The yellow boxes indicate the positions that show a microhomology. Type 2 shows a microhomology of the breakpoint of the left flank with the second base of the negative strand of the right flank. Type 4 shows a microhomology of the second to last base of the excluded part of the right flank, and the last base of the excluded part of the left flank. The microhomology of type 4 is therefore not in the regions that get fused. **e**. The heatmaps represent the sum of all type 2 (top, n = 51 translocations), respective type 4 (bottom, n = 45 translocations), translocation maps derived from the *fog-2* adults (F1) with paternal exposure to IR (90 Gy), excluding translocations with a templated insertion. Color-coded is the ratio of similarity. A darker color shows a higher sequence similarity between the corresponding bases. The numbers on the x- and y-axes are the same as in the explanation in Extended Data Fig. 3D. **f**. Similar to (e) but for WT adults (F1) with parental exposure to IF (90 G y). Type 1 (n = 65 translocations), type 2 (n = 57 translocations) and type 4 (n = 58 translocations) translocation maps derived from the WT adults (F1) with parental exposure to IR (90 Gy), excluding translocation with a templated insertion. **g**. Base composition around the breakpoints of the translocations with a microhomology of length 1 for *fog-2* (left, n = 35) or WT (right, n = 58). Position 0 indicates the break point. Negative numbers are bases not included after the fusion, while positive numbers depict the distance from the fusion site and are included after the fusion. Color-coded are the 4 bases, the shadow in the background shows the distribution of AT, respective CG, for 25.000 permutations. The y-axis shows the percentage of the corresponding base at the given position. **h**. Knockdown of *EV, brc-1, cku-80* and *polq-1* by RNAi on the *fog-2* mutant with or without paternal IR exposure. n indicating biological replicates are shown. Medians with 95% CI are shown (p-values are shown. GLM with logit link function and Tukey multiple comparisons were used). One-way ANOVA with arcsine transformation was also used for the proportion data to confirm the statistical results and included in the full statistical results shown in Supplementary Table 1.

DSBs are mainly repaired by either the error-free HRR or the error-prone non-homologous end joining (NHEJ). In cells deficient in NHEJ or HRR, error-prone TMEJ is crucial for viability^27^. TMEJ involves a microhomology in DNA flanking the breaks and uses DNA close to the breakpoints as a template for de novo synthesis^28^. We analysed the sequence context of the pooled 154 de novo translocations identified in the F_1_ generation of *fog-2* worms. Whereas most translocations showed no insertions in between their breakpoints, around 23% contained at least 1 inserted base pair, and 6% had an insertion of at least 3 bp whose template could be found within ±25 bp around the breakpoints, thereby matching the TMEJ pattern defined in *C. elegans*^28^ (Fig. 3a and Extended Data Fig. 6a). Wild-type samples showed a similar pattern, with 11% templated inserts (Extended Data Fig. 6b,c). This suggests that translocation-inducing error-prone DSB repair could use DNA close to the breakpoints as a template for de novo synthesis preceding end joining.

Given the TMEJ signature, we next tested whether microhomology was present in the sequence around the breakpoints of the de novo translocations. We divided the translocations into four types on the basis of their sequence orientation and identified a microhomology in each of the types. Types 1 and 3 are shown in Fig. 3b and types 2 and 4 are shown in Extended Data Fig. 6d–f. Types 1 and 3 annotate the same translocation—one is annotated from the 5′ direction and the other is from the 3′ direction. For clarity, we refer to both as type 1. We assessed the degree of sequence similarity in an 8-bp window around the breakpoints. The last base pair within the translocation—that is, the breakpoint—is denoted with zero, the three following bases are denoted with positive numbers, and the last four bases that are not included in the translocation are denoted with negative numbers (Fig. 3b right, and Extended Data Fig. 6e). We identified the same patterns in the genomes of wild-type F_1_ worms (Extended Data Fig. 6f). Finally, we investigated the base composition around the breakpoints (Extended Data Fig. 6g). The sequence context of DNA breakpoints is thus reminiscent of the outcomes of error-prone TMEJ.

To corroborate the repair pathway usage, we crossed the mutants of the major DSB repair pathways with *fog-2*: HRR is inactivated in *brc-1* mutants, NHEJ is inactivated in *cku-70* mutants, and TMEJ is inactivated in *polq-1* mutants. HRR defective *brc-1; fog-2* mutants did not further increase the progeny lethality of the P_0_ and F_1_ generations (Fig. 3c), indicating that the paternally inherited DNA damage does not engage the error-free HRR repair pathway. By stark contrast, the TMEJ mutant *polq-1; fog-2* worms were hypersensitive to paternal DNA damage (Fig. 3c), and the rare F_1_ survivors were sterile owing to a germline developmental defect. Depletion of the NHEJ repair protein CKU-70 exacerbated the progeny lethality in the *cku-70; fog-2* ∆m and *cku-70; fog-2* ∆mm compared with *fog-2* single mutants. We further confirmed these observations by using RNA-mediated interference (RNAi) to knock down these repair proteins in the *fog-2* mutant (Extended Data Fig. 6h). These results indicate that the paternal DNA damage is repaired predominantly by TMEJ and partially by NHEJ. Given that TMEJ and NHEJ are both error-prone repair pathways^27–29^, these findings provide an explanation for the chromosomal abnormalities observed in the F_1_ generation.

We next tested whether TMEJ was active in mature sperm or after fertilization with damaged sperm. We sex-separated the *polq-1* mutants and crossed each combination between male and female *fog-2* mutants proficient or deficient (*polq-1*) for TMEJ. Progeny lethality increased to nearly 100% only when *polq-1* females were mated with males treated with ionizing radiation, regardless of paternal *polq-1* status (Fig. 3d), indicating that maternally provided TMEJ is the major route for repair of mature sperm DNA.

**Extended Data Fig. 7:**
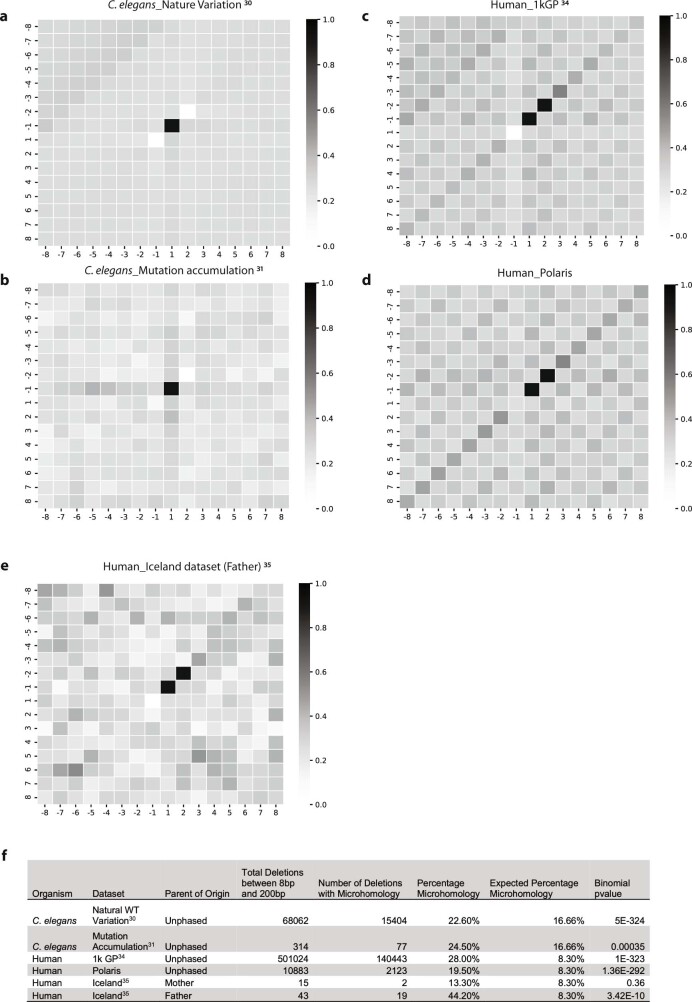
Analysis of public *C. elegans* and human datasets. The heatmaps represent the sum of all base similarities of the microhomology deletion break sites. Color-coded is the ratio of similarity. A darker color shows a higher sequence similarity between the corresponding bases. The x-axis is the right, the y-axis the left flank of the deletion break site. Positive values indicate the flanking sequences, negative values the deleted sequences, i.e. -1 is the first deleted base **a**. The heatmap represents the sum of deletion sites with a length ≤ 200 bp and a 1 bp-microhomology of 540 natural isolates of the CeNDR dataset^30^ (22.6% show a 1 bp-microhomology, expected are 16.66 %, binomial p-value = 5e-324, total deletions n = 68062). **b**. The heatmap shows the sum of deletion sites with a length ≤ 200 bp and a 1 bp-microhomology of samples without mutagen treatment of the public mutation accumulation dataset^31^ (24.5% show a 1 bp-microhomology, 16.66 % expected, binomial p-value = 0.00035, total deletions n = 314). **c**. The heatmap represents the sum of deletion sites with a length ≤ 200 bp and a 2-6 bp microhomology of the human 1000 genome project^34^ (28% show a 2-6 bp-microhomology, expected 8.3%, binomial p-value = 1e-323, total deletions n = 501024). **d**. The heatmap shows the sum of the *de novo* deletion sites with a length ≤ 200bp and a 2-6 bp microhomology of the *de novo* structural variants in the children of trios of the Polaris dataset (https://github.com/Illumina/Polaris) (19.5% show a 2-6 bp-microhomology, expected 8.3%, binomial p-value = 1.36e-292, total deletions n = 10883). **e**. The heatmap shows the sum of the *de novo* paternally-induced deletions with a length ≤ 200 bp and a 2-6 bp microhomology of 1548 trios from Iceland^35^ with known gamete-of-origin (44.2% show a 2-6 bp-microhomology, expected 8.3%, binomial p-value = 3.42e-10, total deletions n = 43). For more details, see Fig. 3E. **f**. Table shows the microhomology results of the re-analysis of the public *C. elegans* and human genome sequencing datasets. The heatmap plots are shown in Extended Data Fig. 7 a-e. Binomial test was used, p-values are shown.

To test whether such a transgenerational effect of TMEJ repair outcome might also occur under physiological conditions, we identified the TMEJ signature in de novo germline mutations in natural *C. elegans* populations and in human germline mutation datasets. TMEJ signatures have previously been identified in five different natural isolates of *C. elegans*^28^. Here we found a highly significant over-enrichment of 1-bp microhomology around the breakpoints of deletion sites in 540 natural isolates of the CeNDR database^30^ (Extended Data Fig. 7a,f). A recent mutation accumulation experiment with wild-type and differentDNA repair mutants involving several mutagens showed a pattern with a substantial microhomology footprint^31^. Re-analysing the deletions of samples treated without any mutagen still showed a significant overrepresentation of the 1-bp microhomology TMEJ footprint (Extended Data Fig. 7b,f). These results are consistent with a requirement for TMEJ for repairing DSBs in germ cells and might therefore be a driver of evolution in unperturbed conditions^32^.

In humans, TMEJ has a 2- to 6-bp microhomology footprint^27,33^. In the 1000 Genomes Project^34^, we found 2- to 6-bp microhomologies in 28% of deletions (Extended Data Fig. 7c,f). These data indicate that the TMEJ footprint is over-represented in natural human variation sites. Similarly, the de novo structural variants in the children of trios of the Polaris dataset (https://github.com/Illumina/Polaris) showed a significant over-representation of microhomology (Extended Data Fig. 7d,f). A recent gamete-of-origin analysis of de novo structural variants in 2,396 families showed that most de novo structural variants are generated in sperm and that children with a sporadic autism spectrum disorder show an increased structural variant count^8^. Moreover, microhomologous structural variants showed an increased ratio for the father as the parent of origin. Similarly, in 1,548 trios from Iceland with known gamete of origin, more de novo deletions were generated in sperm than in oocytes^35^. In our re-analysis of the published mutation calls, the deletions generated in sperm specifically showed a significant microhomology over-representation (Extended Data Fig. 7e,f). TMEJ might thus also have a role in sperm-induced mutagenesis under unchallenged conditions in *C. elegans* as well as in humans.

## Histone H1 and HPL-1 trigger lethality

**Fig. 4:**
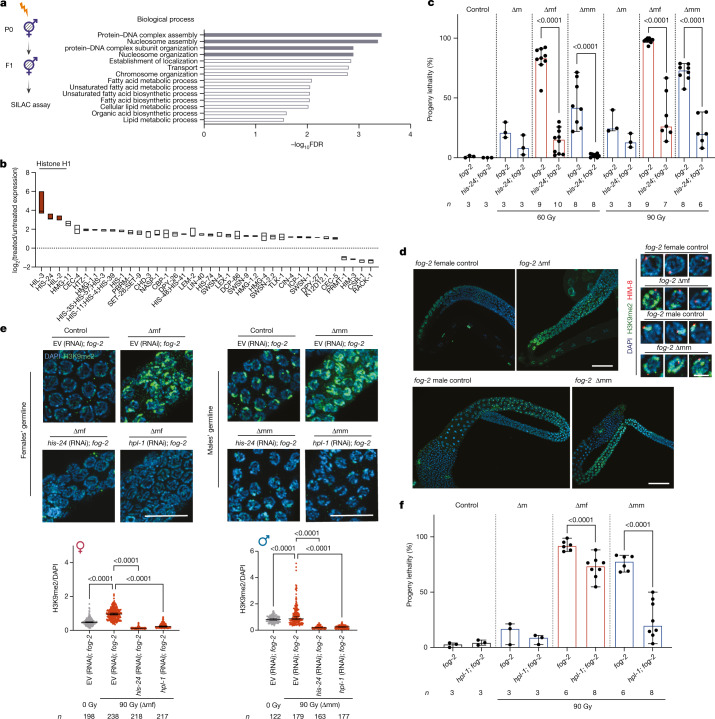
Removal of histone H1 or HPL-1 alleviates lethality inherited from sperm. **a**, Gene Ontology term analysis for biological processes among significantly regulated proteins from a SILAC assay for wild-type worms with or without parental exposure to ionizing radiation. The top four enriched processes are highlighted in grey. FDR, false discovery rate. Values of log_10_FDR larger than 1.3 correspond to FDR < 0.05. **b**, Relative expression of proteins involved in chromosome- or DNA-binding processes upon parental exposure to ionizing radiation. The floating bar shows the range of values, and the centre line represents the median. *n* = 4 biological replicates. **c**, Progeny lethality characterization of the *his-24; fog-2* mutant with or without paternal ionizing radiation irradiation. *n* indicates the number of biological replicates. Data are median ± 95% confidence interval. *P* values are shown. **d**, Left, H3K9me2 (green) and DAPI (blue) staining in the germline of indicated strains. Scale bars, 20 μm. Right, three representative nuclei co-stained with the X chromosome marker HIM-8 (red). Scale bar, 3 μm. **e**, H3K9me2 (green) and DAPI (blue) staining in the germline of indicated RNAi strains with or without paternal ionizing radiation exposure. Scale bar, 10 μm. Bottom, quantification of H3K9me2 signal intensity. *n* indicates cell numbers. Data are median ± 95% confidence interval. *P* values are shown; one-way ANOVA with Bonferroni’s multiple comparisons test. Experiments in **d**,**e** were repeated three times with similar observations. EV, empty vector. **f**, Progeny lethality of *fog-2* and *hpl-1; fog-2* with or without paternal ionizing radiation irradiation. *n* indicates the number of biological replicates. Data are median ± 95% confidence interval. *P* values are shown; GLM with logit link function was used for proportional data (**c**,**f**), and two-tailed *t*-test with arcsine transformation was used to confirm the statistical results. Full statistical analysis is shown in Supplementary Table 1.

**Extended Data Fig. 8:**
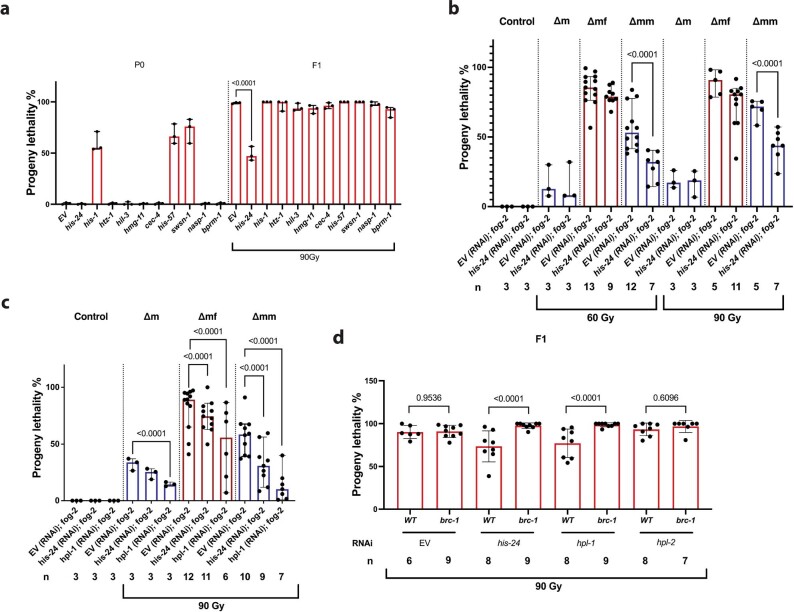
HIS-24 regulates the paternally inherited embryonic lethality. **a**. RNAi screening of ten DNA-associated protein candidates in hermaphrodite WT animals for the paternally inherited transgenerational embryonic lethality. Knockdown of *his-24* significantly reduced the progeny lethality in the F1 generation. n = 3 biological replicates, medians with 95% CI are shown (p-values are shown. GLM with logit link function and Tukey multiple comparisons were used). **b**. Progeny lethality characterization of *EV (RNAi); fog-2* and *his-24 (RNAi); fog-2* with or without paternal IR irradiation (60 Gy and 90 Gy). n indicating biological replicates are shown. Medians with 95% CI are shown (p-values are shown. GLM with logit link function was used). **c**. Progeny lethality characterization of *EV (RNAi); fog-2*, *his-24 (RNAi); fog-2*, and *hpl-1 (RNAi); fog-2* with or without paternal IR irradiation (90 Gy). n indicating biological replicates are shown. Medians with 95% CI are shown (p-values are shown, GLM with logit link function and Tukey multiple comparisons were used). **d**. Knockdown of *EV, his-24, hpl-1* and *hpl-2* in hermaphrodite WT and *brc-1* mutants with parental IR exposure (90 Gy) at the late L4 stage, and characterization of the progeny lethality in the F1 generation. Medians with 95% CI are shown (n indicating biological replicates are shown, p-values are shown. GLM with logit link function and Tukey multiple comparisons were used). One-way ANOVA (**a** and **c**) Two-way ANOVA (**d**) and two-tailed t-test (**b**) with arcsine transformation were also used for the proportion data to confirm the statistical results and are included in the full statistical results shown in Supplementary table 1.

To identify the global changes in the proteome of the F_1_ worms of parents treated with ionizing radiation, we performed stable isotope labelling with amino acids (SILAC)-based proteomics (Fig. 4a) using wild-type hermaphrodites for feasibility. The proteome changes were highly enriched in protein–DNA assembly, nucleosome assembly and nucleosome organization (Fig. 4a), with the majority of the proteins being up-regulated in the F_1_ of worms treated with ionizing radiation, especially the histone proteins (Fig. 4b). The three most highly up-regulated proteins were the linker histone (H1) variants, HIL-3, HIS-24 and HIL-2. Knockdown of *hil-2* by RNAi led to larval arrest regardless of irradiation, whereas *hil-3* RNAi showed only a mild, but not significant, reduction of lethality in the F_1_ progeny. Notably, RNAi knockdown of *his-24* significantly restored the embryonic viability of the wild-type F_2_ generation (Extended Data Fig. 8a).

Consistently, *his-24; fog-2* double mutants significantly suppressed the transgenerational embryonic lethality caused by paternal ionizing radiation exposure (Fig. 4c). However, the rescue effect of HIS-24 depletion was quickly reduced after maintaining this strain for multiple generations, implying that the function of HIS-24 can be gradually compensated by other regulators. To circumvent such an adaptation, we knocked down *his-24* in *fog-2* mutants using RNAi. The viability was significantly restored in the progeny of ∆mm, whereas *his-24* RNAi was insufficient to reduce the high lethality levels in progeny of ∆mf, probably because even one aberrant X chromosome structure is sufficient to prevent the MSCI, thus resulting in lethality (Extended Data Fig. 8b). These data indicate that the inherited paternal DNA damage leads to an increase in the level of histone H1, which is responsible for the embryonic lethality in the F_2_ generation.

Histone H1 is involved mainly in DNA packaging and chromosomal condensation, and functions together with heterochromatin proteins to regulate chromatin mobility, higher-order chromatin structure and transcription^36,37^. Thus the up-regulated histone H1 in the F_1_ generation might be associated with an increase in heterochromatin regions. In non-irradiated *fog-2* mutants, the H3K9me2 heterochromatin mark was weakly but evenly distributed on the chromatin of late pachytene cells of the females and strongly enriched on the X chromosome in the pachytene cells of the males, as it is essential for the X chromosome silencing during meiosis in the male germline (Fig. 4d). In the germline of the F_1_ worms, we found a marked increase in the level of H3K9me2 that was not specifically associated with the X chromosome, but was randomly distributed (Fig. 4d). Depletion of HIS-24 significantly abolished the increased H3K9me2 in the germlines of both *fog-2* ∆mf and *fog-2* ∆mm worms (Fig. 4e).

HPL-1 is essential for the establishment and maintenance of heterochromatin structure. Similar to *his-24* (RNAi); *fog-2*, HPL-1 knocked-down in *fog-2* mutants resulted in the loss of H3K9me2 in the germline of the F_1_ worms (Fig. 4e). Consistently, we detected significant suppression of the progeny lethality of paternally irradiated *hpl-1; fog-2* mutant worms (Fig. 4f). We verified the restoration of viability by *hpl-1* RNAi in *fog-2* mutants (Extended Data Fig. 8c). Our observations suggest that the paternal DNA damage causes increased histone H1 expression, leading to increased heterochromatin formation, which in turn results in embryonic lethality in the F_2_ generation.

## HRR prevents transgenerational lethality

**Fig. 5:**
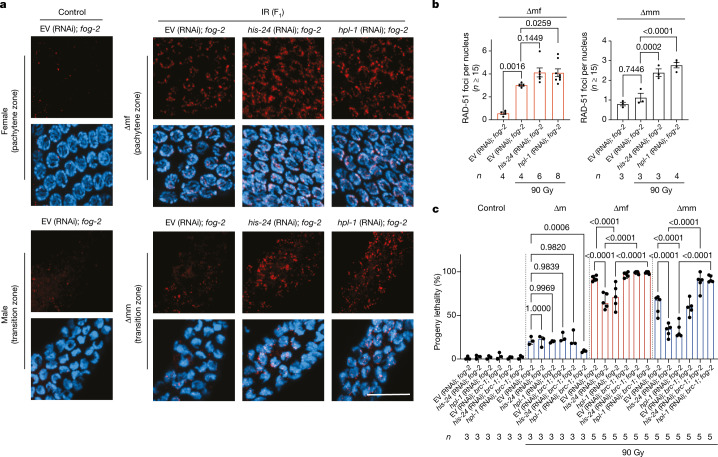
Depletion of histone H1 or HPL-1 triggers HRR to alleviate transgenerational lethality. **a**, RAD-51 immunofluorescence (red) and DAPI staining (blue) in the germlines of EV (RNAi); *fog-2*, *his-24* (RNAi); *fog-2* and *hpl-1* (RNAi); *fog-2* worms with or without paternal exposure to 90 Gy ionizing radiation. Top, pachytene zone of the female germlines. Bottom, transition zone of male germlines. Scale bar, 10 μm. Experiment in **a** was repeated three times with similar observations. **b**, Quantification of RAD-51 foci per nucleus in the germline of females and males, with or without paternal exposure to 90 Gy ionizing radiation. More than 15 nuclei were counted for each germline; *n* represents the number of germlines. Each dot shows the mean of the number of RAD-51 foci per nucleus in one germline. Bars show median ± 95% confidence interval. *P* values are shown; nested one-way ANOVA with Bonferroni’s multiple comparisons test, reflecting the variation between different germlines. **c**, Progeny lethality characterization of empty vector, *his-24* and *hpl-1* RNAi on *fog-2* and *brc-1; fog-2* mutants with or without paternal ionizing radiation irradiation (90 Gy). Control, ∆m: *n* = 3; ∆mf, ∆mm: *n* = 5 biological replicates. Data are median ± 95%confidence interval. *P* values are shown; GLM with logit link function and Tukey multiple comparisons; one-way ANOVA with arcsine transformation was used for the proportional data to confirm the statistical results and are included in the full statistical results (Supplementary Table 1).

Heterochromatization could prevent faithful DSB repair, such as HRR^38,39^. There was a modest increase in RAD-51 foci, which indicate HRR^40^, in the germline of F_1_ worms compared with offspring from non-irradiated worms. Knockdown of *his-24* and *hpl-1* in *fog-2* mutants resulted in significantly increased RAD-51 foci in the germline of ∆mm worms, whereas the germline of ∆mf worms showed a significant, albeit mild, increase in RAD-51 foci in *hpl-1* (RNAi); *fog-2* mutants (Fig. 5a,b). The increase in RAD-51 foci was most obvious in the pachytene zone of the ∆mf germline and in the transition zone of the ∆mm germline.

To assess whether the rescue effect in the worms devoid of HIS-24 and HPL-1 is owing to the activation of HRR, we knocked down *his-24* and *hpl-1* in the *brc-1; fog-2* double mutant. The rescue of progeny lethality upon *his-24* and *hpl-1* depletion by RNAi was completely abolished in *fog-2; brc-1* mutants, indicating that HRR mediates the restored viability of the F_1_ progeny upon H1 depletion (Fig. 5c). We confirmed the requirement of HRR for the ability of HIS-24 and HPL-1 depletion to restore viability in hermaphrodite wild-type and *brc-1* mutants (Extended Data Fig. 8d). Although the depletion of BRC-1 completely abolished the progeny viability of the H1-depleted F_1_ offspring, it did not affect the progeny viability of the P_0_ generation, indicating that the H1-mediated DNA repair restriction mainly affects the F_1_ germline.

**Fig. 6:**
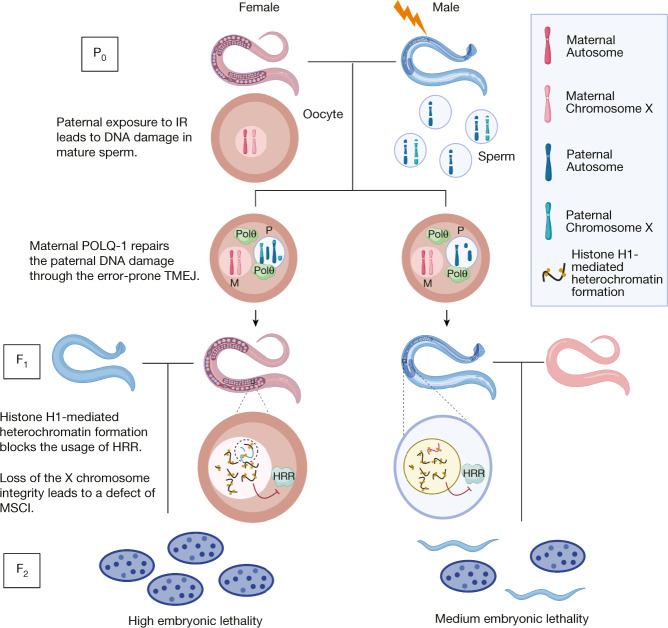
Schematic model for the transgenerational effect of the paternal exposure to ionizing radiation. Ionizing radiation (IR) exposure of male *C. elegans* leads to DNA DSBs in the mature sperm, which carries the fragmented DNA into the fertilized (un-irradiated) oocyte. The maternal oocyte provides the TMEJ machinery to repair the paternal DNA damage, resulting in various chromosomal aberrations. In the germline of the F_1_ offspring, linker histone H1-mediated heterochromatin structures accumulate in the chromatin of the germ cells, which prevents the DNA DSBs from being detected and repaired by the error-free HRR. Specifically in the female F_1_ (∆mf), X chromosome translocations and fragmentations prevent MSCI, thus further aggravating the embryonic lethality in the progeny. Together, paternal exposure to ionizing radiation leads to transgenerational embryonic lethality via heterochromatization-restricted DNA repair access.

We conclude that the paternally inherited DNA damage leads to increased histone H1 and heterochromatin formation, which in turn prevents the F_1_ generation from using the faithful DSB repair machinery. Removal of the heterochromatin barrier facilitates the engagement of the error-free HRR and significantly rescues the transgenerational embryonic lethality caused by paternal ionizing radiation irradiation (Fig. 6).

## Discussion

The determination of the transgenerational consequences of parental exposure to ionizing radiation has been limited mainly because the mechanisms underlying this process have remained unknown. Studies in animals have provided evidence that paternal exposure to ionizing radiation could result in compromised viability, fertility and genome stability in the offspring^41^. Bovine oocytes that are fertilized with ionizing radiation-treated mature sperm display chromosomal fragmentation and translocations of the paternal chromosomes during early embryogenesis, resulting in aneuploidy and unequal chromosome segregation^42^. Such genetic mosaicism is the leading cause of the high miscarriage rate and low implantation rate in in vitro fertilization^43,44^. Viable F_1_ offspring of irradiated male mice display increased genomic instability and increased incidence of cancers^45,46^. The specific vulnerability of mature sperm is consistent with the correlation between the increased risk of malignant disease in children and paternal radiation exposure around the time of conception—that is, when mature sperm has been exposed to radiation^14,16^. Consistently, germline hypermutation in humans has recently been associated with paternal exposure to chemotherapeutic agents before conception^21^, which might increase the risk of genetic disease in the offspring^22^.

The specific vulnerability of mature sperm is explained by its highly compacted chromatin structure precluding DSB repair, necessitating repair by maternal factors after fertilization. Depletion of the HRR and NHEJ factors in mice could significantly increase chromosomal structural aberrations in early embryos carrying paternal DSBs^23^. We found that in *C. elegans*, maternal TMEJ is the major DSB repair mechanism acting on the paternal genome upon fertilization. The de novo germline structural variants generated under physiological conditions also show the TMEJ signature and in humans is particularly enriched in paternally generated DNMs^5,47,48^. From an evolutionary perspective, unlike the stable maternal genome, the unstable paternal genome contributes greatly to the genetic diversity of a species. TMEJ appears to have a major role in shaping genome evolution, as its signature is present both in *C. elegans* variants^32^ and in paternal human structural variants. It is thus tempting to speculate that the mechanisms governing the engagement of maternal TMEJ in the paternal genome thereby contribute to genetic diversification.

Our observation that the genome instability observed in the progeny of the irradiated males is driven by changes in the epigenome provides a rationale for developing potential therapeutic approaches to prevent the detrimental heritable consequences of parental radiation exposure. Heterochromatin inhibitors or H3K9 methyltransferase inhibitors^49^ might potentially be considered for promoting HRR in the progeny carrying paternal genome instability. In addition, our work further emphasizes that the paternal genome in mature sperm is particularly vulnerable, potentially giving rise to genome instability in the progeny, increasing the need for protection from mutagenic exposure particularly in the two months period pre-conception. In sum, our work elucidates a previously unknown mechanism underlying the transgenerational effect of paternal exposure to radiation and mandates further evaluation of the transgenerational effects of paternal DNA damage in humans.

## Methods

### *C. elegans* strains

All strains were maintained based on standard conditions at 20 °C. Strains used were N2 (Bristol; wild type), CB4108 *fog-2(q71)*, BC784 *spe-8(hc50)*, RB1067 *his-24(ok1024)*, MT13971 *hpl-1(n4317)*, DW102 *brc-1(tm1145)*, FX1524 *cku-70(tm1524)*, FX2026 *polq-1(tm2026)*, BJS1017 *his-24(ok1024); fog-2(q71)*, BJS1018 *hpl-1(n4317); fog-2(q71)*, BJS1019 *brc-1(tm1145); fog-2(q71)*, BJS1020 *cku-70(tm1524); fog-2(q71)*, BJS1021 *polq-1(tm2026); fog-2(q71)*.

### Measurement of ionizing radiation-induced progeny lethality

For the feminized mutants, synchronized L4 females and males were separated and maintained overnight. On the second day, the adult females or males either remained untreated or were irradiated with the indicated dose of ionizing radiation inflicted by a caesium 137 irradiation source (Biobeam GM 8000, Eckert & Ziegler, Gamma-Service Medical). Afterwards, ≥3 irradiated adult worms and ≥3 non-irradiated opposite-sex adults were immediately transferred to 3 new plates served as 3 biological replicates and allowed to lay eggs for 2 h. Then the males were removed and left the females to continue egg-laying for another 4 h. The females were then removed and the number of eggs was counted. The number of surviving progeny was characterized 24 h later to examine the progeny lethality of the P_0_ generation. Then, we transferred ≥3 surviving male progeny (F_1_) or ≥3 surviving female progeny (F_1_) to new plates, serving as one biological replicate, and placed them with the three untreated opposite-sex adults and allowed them to lay eggs for one day. At least three biological replicates were included in each experiment. The adults were then removed and the number of laid eggs was counted. Twenty-four hours later, the surviving progenies were counted to characterize the progeny lethality of the F_1_ generation.

For the hermaphrodite worms, synchronized hermaphrodite late L4 were separated from the rest of the worms by picking, and ≥3 late L4 hermaphrodites were either untreated or irradiated with the indicated dose of ionizing radiation. Three irradiated hermaphrodites were transferred to 3 separate plates as 3 biological replicates and allowed to lay eggs for 6 h. The adults were then removed and the number of eggs was counted 24 h later, the hatched progeny were quantified as the progeny lethality of the P_0_ generation. The surviving worms were transferred to three plates and allowed to lay eggs for one day. The adults were removed and the progeny lethality of the consequent generations was quantified 24 h later.

### Developmental assay

Synchronized L1 worms of control and paternally treated F_1_ were generated via standard hypochlorite treatment. Arrested L1 worms were placed on NGM agar plates with OP50, fed with bacteria and incubated at 20 °C for 48 h. Then the larval stage of worms was characterized under a Zeiss discovery.V8 microscope. For each experiment, >30 L1 larvae were included for each replicate, *n* = 3 biological replicates were used.

### Quantification of germ cell apoptotic corpses

Day-1 adult female worms were immobilized using 5 mM levamisole (AppliChem A431005) and mounted on a 2% agarose pad on a microscope slide. The number of apoptotic corpses was scored via Nomarski DIC microscopy on a Zeiss Axio Imager M1/2 based on the refractive morphological changes occurring in apoptotic germ cells within the gonad loop^50^.

### RNAi treatment

RNAi feeding clones were obtained from the library of J. Ahringer. The *E. coli* feeding strain HT115 (DE3) with RNAi clones were cultured with LB medium containing ampicillin (100 μg ml^−1^) overnight. IPTG (1 mM) was added to the culture for the induction of RNAi product before seeded on RNAi agar plates (NGM agar with ampicillin and IPTG). For the RNAi feeding assay, >30 synchronized L1 larvae as P_0_ generation were placed on the RNAi agar plates seeded with *E. coli* feeding strain HT115 (DE3) containing specific RNAi or empty vector control. Three days later, adult males and females were separated and transferred to fresh RNAi plates for maintaining the RNAi efficiency until further experiments were performed. The subsequent experiments were performed as described in ‘Measurement of ionizing radiation-induced progeny lethality’.

### Immunofluorescence staining

Adult worms were picked from plates and transferred to a drop of M9 buffer onto a 0.3% polylysine-treated three-well slide (3 × 14 mm printed wells slides from Fisher Scientific). Germline dissection was carried out with two syringe needles, followed by fixation with 3.7% formaldehyde for 1 h. Then, a 24 × 24 mm coverslip was placed onto the drop, and the slide was left in a −80 °C freezer for 10 min to perform the freeze-cracking procedure. Then the slide was quickly transferred to −20 °C methanol for less than 1 min. For visualizing the nuclei, slides were washed once with PBS and once with PBST (0.2% Tween in PBS) and mounted with DAPI Fluoromount-G mounting medium (Southern Biotech) and sealed with nail polish. For the other staining, after fixation, slides were washed 1 time with 1× PBS and 2 times with 1× PBT (0.5% Triton X-100 in PBS). To improve the signal quality, slides were first blocked for 20 min with Image-iT FX signal enhancer (Thermo Fisher) before blocking with 1× PBT containing 10% donkey serum for another 20 min. Afterwards, primary antibodies diluted with 1× PBT containing 5% donkey serum were applied to the slides and incubated at 4 °C overnight. After washing 3 times with 1× PBT, the slides were incubated with secondary antibodies diluted with 1× PBT at 37 °C for 30 min. Then slides were washed with 1× PBT 3 times, mounted with DAPI Fluoromount-G mounting medium (Southern Biotech) and sealed with nail polish. Slides were stored at 4 °C in the dark before imaging.

Primary antibodies used for immunofluorescence staining were rabbit polyclonal anti-phospho-RNAPII (Ser2) antibody (Thermo Fisher, A300-654A; dilution 1:500 in PBT); mouse monoclonal anti-H3K9me2 antibody (Abcam, ab1220; dilution 1:100 in PBT); rabbit polyclonal anti-HIM-8 (Novus Biologicals, 41980002; dilution 1:100 in PBT); rabbit anti-RAD-51 antibody (a gift from the laboratory of A. Gartner; dilution 1:2,000 in PBT). Secondary antibodies used were AlexaFluor 488 donkey anti-mouse IgG (Thermo Fisher, A21202; dilution 1:500 in PBT) and AlexaFluor 594 donkey anti-rabbit IgG (Thermo Fisher A21207; dilution 1:500 in PBT).

Fluorescence images for quantification were taken with a Zeiss Meta 710 confocal laser scanning microscope. For quantification, fixed exposure time was set for different treatments and strains. For H3K9me2, RNAPII p-Ser2 and RAD-51 staining, *z*-stack images were acquired with Zeiss Meta 710 confocal microscope, and the H3K9me2 and RNAPII p-Ser2 signal intensity and the foci number of RAD-51 foci per nucleus were quantified with Imaris x64 9.1.2 software. Fluorescence intensities were normalized to DAPI signal.

### SILAC assay

The stable isotope labelling procedure was described in a previous study^51^. In brief, ET505 *E. coli* (lysine auxotrophy, from Coli Genetic Stock Center) were grown in M9 minimal medium (Na_2_HPO_4_ 5.8 g l^−1^, KH_2_PO_4_ 3 g l^−1^, NaCl 0.5 g l^−1^, NH_4_Cl_2_ 1 g l^−1^, glucose 0.2% (w/v), MgSO_4_ 1 mM, thiamine 0.01% (w/v) and 40 µg ml^−1^ ^13^C_6_-labelled lysine (Cambridge isotope laboratory) or 40 µg ml^−1^ normal l-lysine, and incubated at 37 °C overnight to reach *A*_600_ = 1. Bacteria were concentrated to *A*_600_ = 50 and seeded to NGM-N plates (3 g of NaCl and 12 g of agarose dissolved in 970 ml deionised water).

Synchronized embryos generated by hypochlorite treatment were hatched and arrested in M9 buffer, and then L1 worms were transferred to NGM-N plates seeded with heavy isotope labelled lysine (heavy lysine)- or normal lysine (light lysine)-labelled ET505 *E. coli*. Worms were fed with labelled bacteria for two generations to reach the incorporation rate >97%, and then picked the late L4 stage worms to irradiate with ionizing radiation or mock-treated. Four replicates were included in this experiment, as two of them were the ionizing radiation-treated heavy lysine group and mock-treated light lysine group, whereas the other two replicates were the mock-treated heavy lysine group and ionizing radiation-treated light lysine group.

Equal numbers of the F_1_ adult worms were washed off from heavy lysine plates and light lysine plates with M9 buffer and combined. After removing the M9 buffer, lysis buffer was added to the worm pellet (6 M guanidinium chloride (GuCl), 10 mM TCEP, 40 mM CAA, 100 mM Tris-HCl). Heat the sample at 95 °C for 10 min and sonicate the sample with Bioruptor (30 s sonication, 30 s break, 10 cycles, high performance). Heating and sonication were repeated once more. Then samples were centrifuged at 20,000*g* for 20 min, and the supernatant was collected. Five microlitres of protein solution was diluted with 20 mM Tris to reduce the concentration of guanidinium chloride to below 0.6 M. 50 mM TEAB was added to dilute the samples to 100 µl and then 1 µg Lys-C was added for incubation at 37 °C for 4 h. Samples were further diluted with 180 µl TEAB and treated with 2 µg Lys-C at 37 °C overnight. Enzyme digestion was stopped by adding formic acid to 1%, and the sample purification by StageTip was carried out according to CECAD/CMMC Proteomics Core facility’s standard protocol (https://www.proteomics-cologne.com/protocols). The mass spectrometry proteomics data have been deposited to the ProteomeXchange Consortium via the PRIDE partner repository with the dataset identifier PXD031873.

### Single-worm WGS

Single L4 male and female *fog-2*, and hermaphrodite wild-type with indicated treatment were transferred to plates with UV-killed OP50 bacteria, in order to reduce the contamination of bacterial DNA. On the second day, a single adult worm was picked to 10 µl of M9 buffer, then the samples were frozen at −80 °C. DNA was extracted following the standard Illumina DNA preparation protocol. Libraries were prepared for sequencing using the standard Illumina protocols. In brief, 120 ng of genomic DNA was tagmented with adaptor sequence using bead-linked transposomes. Tagmented DNA was amplified by PCR for 5 cycles. Libraries were sequenced on the Illumina HiSeq 2500 following the manufacturer’s protocols. The data have been deposited with links to BioProject accession number PRJNA826255 in the BioProject database.

### Telomere FISH

Telomere FISH was carried out by a modified protocol^52^. Twenty adult females or males were transferred to a drop of M9 buffer onto a 0.3% polylysine-treated 3-well slide (3 × 14 mm printed well slides; Fisher Scientific). Germline dissection was carried out with two syringe needles, followed by fixation with 3.7% formaldehyde for 1 h. Then a 24 × 24 mm coverslip was placed onto the drop, and the slide was left in a −80 °C freezer for 10 min to perform the freeze-cracking procedure. Then the slide was quickly transferred to −20 °C methanol for less than 1 min. The slides were washed once with 1× PBS and incubated in permeabilization buffer (0.5% Triton X-100 in 1× PBS) for 1 h at room temperature followed by a wash in 1× PBS. Then slides were quickly washed with 0.01 N HCL followed by a wash with 0.1 N HCL for 2 min. To prevent unspecific binding of the FISH probe, 50 µg ml^−1^ RNase A solution (10 µg ml^−1^ RNase A in 1× PBS) was added to the slide and incubated at 37 °C for 45 min. Afterwards, slides were washed 2 times with 2× SSC. For pre-hybridization, 50 µl of pre-hybridization solution (2× SSC, 50% formamide) was added on the slides and incubated in a humid chamber for 2 h at room temperature. Then the FISH probe (PNA-FISH TTAGGC telomeric probe, Panagene, resuspended to 100 µM, fluorophore: Cy3) was diluted as 1:500 in hybridization buffer (2× SSC, 50% formamide, 10% (w/v) dextran sulfate, 50 µg ml^−1^ heparin, 100 µg ml^−1^ sheared salmon sperm DNA). After pre-hybridization, the solution was removed from the slide as much as possible, then 50 µl of FISH probes were added to the sample and covered with Frame-Seal in situ PCR and Hybridization Slide Chamber (Bio-Rad SLF0601), then the slides were incubated over-night at 37 °C. On the second day, the samples were denatured for 5 min at 80 °C and continued incubating at 37 °C for 2 days. Afterwards, the hybridization chambers were removed, and slides were washed in 2× SSC for 5 min at room temperature. To fixate the staining, the slides were washed 3 times in preheated 2× SSC at 37 °C, and twice in preheated 0.2× SSC at 55 °C. As the last step, slides were washed in 1× PBT for 10 min at room temperature and mounted by 7 µl Vectashield mounting medium containing DAPI (Vector Laboratories H-1200-10).

Staining images were taken with a Zeiss Meta 710 confocal laser scanning microscope was used. To visualize the telomeric signal, *z*-stack images were acquired with a Zeiss Meta 710 confocal microscope, and the *z*-stack pictures were processed and the number of DNA fragments was counted with Image J/Fiji v2.3.0/1.53f.

### Public datasets

The following public datasets have been re-analysed: The deletions of the hard-filtered variants of the 20220216 CeNDR 29 release^30^ were downloaded from https://www.elegansvariation.org/data/release/20220216. The data for the *C. elegans* mutation accumulation experiment were downloaded from the supplementary data from Volkova et al.^31^. The filtered hg38 SNV_INDEL_SV_phased_panel.vcf files for all chromosomes from the 20220422 release of the 1000 Genomes Project^34^ were downloaded from http://ftp.1000genomes.ebi.ac.uk/vol1/ftp/data_collections/1000G_2504_high_coverage/working/20220422_3202_phased_SNV_INDEL_SV/. The hg38 illumina-polaris-v2.1-sv-truthset structural variants (https://github.com/Illumina/Polaris) were downloaded from https://s3-us-west-1.amazonaws.com/illumina-polaris-v2.1-sv-truthset/all_merge.vcf.gz. The processed hg38 variants of the 1,548 trios from Iceland including gamete-of-origin analysis were downloaded from the supplementary data from Jónsson et al.^35^.

### Bioinformatics analysis

#### Gene set enrichment analysis

The enrichment analysis for chromosomal gene distributions was done in R v3.6.3 with the GSEA function of clusterProfiler v3.14.3^53^ was used with maxGSSize = 20000 and nPerm = 20000.

#### WGS preprocessing

The fastq files were preprocessed with Fastp v0.20.0^54^, and mapped with BWA-0.7.17^55^ with the parameters bwa mem -M -K 100000000, and the reference genome ce11. The mapped files were converted to BAM and sorted with samtools v1.6^56^, and duplicated reads were removed with GATK v4.1.0.0 MarkDuplicates^57^.

#### Structural variant calling

Structural variants were called with Manta v1.6.0^58^ and only structural variants that passed all of the Manta quality filters were used. To find structural variants that are newly induced in the F_1_ generation, structural variants that overlapped with any structural variant of any P_0_ sample were filtered out (full structural variation sites with or without filtering in Supplementary Table 3). Repeat regions are difficult to map and identify, we therefore deleted any insertion–deletion mutant within a repeat region, or any translocation for which both break points overlapped with the same repeat class. Manta calls translocations in both directions as two break points including a position confidence interval. To avoid duplicates, we filtered translocations that had the same start and end break point within the respective confidence interval in any combination.

#### Translocation types

A translocation is called as two break points and can appear in four different ways in a VCF file. The reference sequence s is replaced by the sequence t after the fusion to position p, respective before the fusion at position p. This can happen in four ways:

- Type 1: t[p[ The genomic location extending right from the position p is fused after t. In other words, these are fusions between the 3′ sense strand with the 5′ sense strand.
- Type 2: t]p] The reverse component of the genomic location left of the position p is fused after t. In other words, these are fusions between the 3′ sense strand and the 5′ anti-sense strand.
- Type 3:]p]t The genomic location extending left from the position p is fused before t. This is the same as Type 1.
- Type 4: [p[t The reverse component of the genomic location extending right from the position p is fused before t. In other words, these are fusions between the 3′ anti-sense strand and the 5′ sense strand.

See https://github.com/samtools/hts-specs/blob/master/VCFv4.1.pdf for further details.

#### Circos plots

The library circlize v0.4.12^59^ in R v3.6.3 was used to generate circos plots.

#### Templated insertions with distribution

The inserted sequence between the break points was searched within ±25 bp around the break points in both directions, in the normal orientation, as well as in the reverse, complement, and reverse complement orientation. Insertions ≥3 bp for which a template could be found within ±25 bp were called templated insertions, while any other insertion was classed as miscellaneous.

#### Microhomology with permutations

For each translocation 8 bp surrounding both break sites (that is, 4 bp upstream and 4 bp downstream of both break sites) were compared in an 8 × 8 grid (that is, each of the surrounding bases is compared to every other base). Matching bases were scored 1 and nonmatching bases were scored 0. One map therefore contains a 1 for each of the 64 combinations that have the same base, and 0 otherwise. The heat maps shown in the figure contain the sum of all such respective heatmaps divided by the total number of translocations. For each of the four translocation classes a separate microhomology was calculated. For type 1 translocations the left and right flank are both 5′ to 3′ on the sense strand. The left flank of type 2 translocations is the 5′ to 3′ sense strand, while the right flank is the reverse complement sequence. For type 4 translocations the left flank is the reverse complement sequence, while the right flank is the 5′ to 3′ sense strand sequence.

To calculate the significance of individual bins a permutation test was done. For each permutation the same number of translocations (of the same type) as in the original heatmap was randomly distributed on the genome to calculate the microhomology ratios for each of the 64 bins. To calculate a *P* value a permutation test was calculated with 100,000 permutations. To calculate the adjusted *P* value for the 64 bins statsmodels v0.11.1^60^ multipletests methods with the parameter method=’fdr_bh’ in Python 3.6^61^ was used.

#### Base composition

For all translocations with a microhomology of length 1 (*n* = 35 for *fog-2*, and *n* = 58 for wild type) the base composition for 8 bp around the break points was calculated. For each of the 8 positions the percentage of A, C, T and G was calculated. To be able to compare it to a random background distribution we sampled the same number of positions—that is, 35 for *fog-2* and 58 for wild type—and calculated the average percentage of each A, C, T, and G for 25.000 such permutations.

### Analyses of public *C. elegans* datasets

#### CeNDR

The deletions of the hard-filtered variants of the 20220216 CeNDR^30^ release were downloaded and further filtered for deletions between 8 and 200 bp. Deletions for which both break sites were annotated within the same repeat class were removed. Each deletion got categorized into non-homology, that is, no matching base at the break sites, microhomology—that is, exactly one matching base at the break sites, and macro-homology—that is, more than one matching base. By chance we would expect 75% of break sites to be non-homologous, 16.66% microhomologous, and 8.33% macro-homologous. The over-representation of microhomologous deletions sites were calculated with the binomial test function binom_test in Python’s Scipy-v1.5.1 package. The over-represented microhomologous variants were used in the heat map as described above for a 16 × 16 grid.

#### Mutation accumulation

The data for the mutation accumulation experiment were downloaded from the supplementary data from Volkova et al.^31^. Deletions for which both break sites were annotated within the same repeat class were removed, and only deletions with a length between 8 and 200 bp were considered for the subsequent analysis. The statistics and heat map were calculated as described above.

### Analyses of public human datasets

#### 1000 Genomes Project

The filtered hg38 SNV_INDEL_SV_phased_panel.vcf files for all chromosomes from the 20220422 release of the 1000 Genomes Project^34^ were downloaded and further filtered for deletions between 8 and 200 bp. Since the microhomology footprint of humans is 2–6 bp, we defined the 3 categories different from *C. elegans*. 0–1 bp homology is expected by chance in 75% + 16.66% = 91.66% of break sites. Microhomology—that is, 2–6 bp homology, in 8.325% of break sites, and macrohomology in 1/12288 ≈ 0.00008% of break sites. The statistics and heat map were otherwise calculated as described above.

#### Polaris

The hg38 illumina-polaris-v2.1-sv-truthset structural variants (https://github.com/Illumina/Polaris) were downloaded and filtered for deletions that had a PASS in the quality column. To focus on de novo deletions, any deletion that overlapped with either parent got filtered out, and only deletions between 8 and 200 bp were considered for the subsequent analysis. The statistics and heat map were calculated as described above.

#### Iceland trios

The processed hg38 variants of the 1548 trios from Iceland including gamete-of-origin analysis were downloaded from the supplementary data from Jónsson et al.^35^. Only deletions between 8 and 200 bp for which the gamete of origin was available were considered. The statistics and heat map were calculated as described above separately for deletions coming from the mother and father.

### Data presentation and statistical analysis

All the data and statistical significances were analysed using the GraphPad Prism 7 software package (GraphPad) and R studio. For the proportion data shown in this paper, GLM with logit link function (R v4.0.2 and emmeans v1.5.2 (https://cran.r-project.org/web/packages/emmeans/index.html)) and ordinary ANOVA with arcsine transformed value (arcsine transformation equation: *Y* = arcsin(√(*Y*/*n*)) × 180/π) were both applied to confirm the significance of the observations, and the full statistic results are shown in the Supplementary Table 1. In addition, a QQ plot was attached for the ANOVA analysis to assess the normal distribution of the transformed value. Only the *P* values calculated from the GLM method are shown in the figures. Statistical methods, *P* values, sample size information and error bar descriptions are reported in the figure legends. Randomization was not applied because the group allocation was guided based on the genotype of the respective mutant worms. Worms of a given genotype were nevertheless randomly selected from large strain populations for each experiment without any preconditioning. Blinding was not applied as the experiments were carried out under highly standardized and predefined conditions such that an investigator-induced bias can be excluded. For progeny lethality characterization and staining quantifications, median with 95% confidence interval was used as these data types contain outliers.

### Reporting summary

Further information on research design is available in the Nature Portfolio Reporting Summary linked to this article.

## Online content

Any methods, additional references, Nature Portfolio reporting summaries, source data, extended data, supplementary information, acknowledgements, peer review information; details of author contributions and competing interests; and statements of data and code availability are available at 10.1038/s41586-022-05544-w.

## Supplementary information

Reporting Summary

Supplementary Table 1 Complete statistical analyses.

Supplementary Table 2 Number of the new translocations in the F_1_ offspring per sample.

Supplementary Table 3 Complete list of called chromosomal structural variation sites before and after filtering with P_0_.

Supplementary Table 4 The results of the proteomic GSEA for chromosomal enrichment.

## Data Availability

The single-worm WGS data have been deposited in the BioProject database under accession number PRJNA826255. The mass spectrometry proteomics data have been deposited to the ProteomeXchange Consortium via the PRIDE partner repository with the dataset identifier PXD031873.

## Code availability

Custom code used in this study is available from https://github.com/Meyer-DH/swWGS_Custom_Code.
