# Supplementary material for: Inheritance of paternal DNA damage by histone-mediated repair restriction
Source: Nature. 2022 Dec 21;613(7943):365–74. doi: 10.1038/s41586-022-05544-w (PMC9834056; doi:10.1038/s41586-022-05544-w)
Supplement: Supplementary file 1 — Reporting Summary [file 41586_2022_5544_MOESM1_ESM.pdf]

nature portfolio

Corresponding author(s): Björn Schumacher

Last updated by author(s): Oct 19, 2022

Reporting Summary

Nature Portfolio wishes to improve the reproducibility of the work that we publish. This form provides structure for consistency and transparency in reporting. For further information on Nature Portfolio policies, see our Editorial Policies and the Editorial Policy Checklist.

Statistics

For all statistical analyses, confirm that the following items are present in the figure legend, table legend, main text, or Methods section.

n/a	Confirmed	
☐	☒	The exact sample size (*n*) for each experimental group/condition, given as a discrete number and unit of measurement
☐	☒	A statement on whether measurements were taken from distinct samples or whether the same sample was measured repeatedly
☐	☒	The statistical test(s) used AND whether they are one- or two-sided<br>*Only common tests should be described solely by name; describe more complex techniques in the Methods section.*
☐	☒	A description of all covariates tested
☐	☒	A description of any assumptions or corrections, such as tests of normality and adjustment for multiple comparisons
☐	☒	A full description of the statistical parameters including central tendency (e.g. means) or other basic estimates (e.g. regression coefficient) AND variation (e.g. standard deviation) or associated estimates of uncertainty (e.g. confidence intervals)
☐	☒	For null hypothesis testing, the test statistic (e.g. *F*, *t*, *r*) with confidence intervals, effect sizes, degrees of freedom and *P* value noted<br>*Give P values as exact values whenever suitable.*
☒	☐	For Bayesian analysis, information on the choice of priors and Markov chain Monte Carlo settings
☒	☐	For hierarchical and complex designs, identification of the appropriate level for tests and full reporting of outcomes
☒	☐	Estimates of effect sizes (e.g. Cohen's *d*, Pearson's *r*), indicating how they were calculated

Our web collection on statistics for biologists contains articles on many of the points above.

Software and code

Policy information about availability of computer code

Data collection	Progeny lethality was examined with stereomicroscope (Leica M80). Staining images were taken by Zeiss Meta 710 confocal laser scanning microscope.
Data analysis	Progeny lethality data were analyzed with GraphPad Prism 7 software package, and Immunofluorescence images were analyzed with Imaris x64 9.1.2 software. Z-stack pictures were processed and the number of DNA fragments was counted with Image J/Fiji v2.3.0/1.53f.<br>The fastq files were preprocessed with Fastp v0.20.0, and mapped with BWA-0.7.17 with the parameters bwa mem -M -K 100000000, and the reference genome ce11. The mapped files were converted to BAM and sorted with samtools v1.6, and duplicated reads were removed with GATK v4.1.0.0 MarkDuplicates. Structural variants (SV) were called with Manta v1.6.0 and the resulting VCF files were further processed and analysis with custom scripts in Python 3.6.10 that are deposited at https://github.com/Meyer-DH/swWGS_Custom_Code<br>Binomial tests for the over-representation of microhomologous deletion sites were calculated with Python's Scipy-v1.5.1 . Adjusted p-values for the permutation analyses were done with Python's statsmodels v0.11.1 .<br>Circos plots were generated with circlize v0.4.12 in R v3.6.3.<br>The GSEA was done with clusterProfiler v3.14.3 in R v3.6.3.<br>The GLM statistics were calculated in R v.4.0.3 and emmeans v. 1.5.2.

For manuscripts utilizing custom algorithms or software that are central to the research but not yet described in published literature, software must be made available to editors and reviewers. We strongly encourage code deposition in a community repository (e.g. GitHub). See the Nature Portfolio guidelines for submitting code & software for further information.

Data

Policy information about availability of data

All manuscripts must include a data availability statement. This statement should provide the following information, where applicable:

- Accession codes, unique identifiers, or web links for publicly available datasets
- A description of any restrictions on data availability
- For clinical datasets or third party data, please ensure that the statement adheres to our policy

Proteomics data have been deposited to the ProteomeXchange Consortium via the PRIDE 65 partner repository (http://proteomecentral.proteomexchange.org/cgi/GetDataset?ID=PXD031873) with the dataset identifier PXD031873. The sequencing data have been deposited with links to BioProject accession number PRJNA826255 in the BioProject database https://www.ncbi.nlm.nih.gov/bioproject/PRJNA826255.

The following public datasets have been re-analysed:

The deletions of the hard-filtered variants of the 20220216 CeNDR 29 release were downloaded from: https://www.elegansvariation.org/data/release/20220216

The data for the C. elegans mutation accumulation experiment were downloaded from the supplementary data from Volkova et al. : https://static-content.springer.com/esm/art%3A10.1038%2Fs41467-020-15912-7/MediaObjects/41467_2020_15912_MOESM9_ESM.zip

The filtered hg38 SNV_INDEL_SV_phased_panel.vcf files for all chromosomes from the 20220422 release of the 1000 genomes project were downloaded from http://ftp.1000genomes.ebi.ac.uk/vol1/ftp/data_collections/1000G_2504_high_coverage/working/20220422_3202_phased_SNV_INDEL_SV/

The hg38 illumina-polaris-v2.1-sv-truthset structural variants were downloaded from https://s3-us-west-1.amazonaws.com/illumina-polaris-v2.1-sv-truthset/all_merge.vcf.gz

The processed hg38 variants of the 1548 trios from Iceland including gamete-of-origin analysis were downloaded from the supplementary data from Jónsson et al.: https://static-content.springer.com/esm/art%3A10.1038%2Fnature24018/MediaObjects/41586_2017_BFnature24018_MOESM2_ESM.zip

Field-specific reporting

Please select the one below that is the best fit for your research. If you are not sure, read the appropriate sections before making your selection.

- [x] Life sciences
- [ ] Behavioural & social sciences
- [ ] Ecological, evolutionary & environmental sciences

For a reference copy of the document with all sections, see nature.com/documents/nr-reporting-summary-flat.pdf

Life sciences study design

All studies must disclose on these points even when the disclosure is negative.

Sample size	Sample size was determined empirically or based on the similar data reported in other scientific publications (Greer et al. 2010, Rinaldo et al. 2002, Maremonti et al. 2019). The embryonic lethality assay we performed requires a high mating rate. In order to increase the chance of successful mating, we included more than 3 females and males (number of worms was separately indicated in figure legends) on single plates, and >=3 plates were included for each condition.
Data exclusions	No data were excluded
Replication	For all embryonic lethality assay, at least three biological replicates were included. Each replicate includes more than 3 female worms and 3 male worms or 3 hermaphrodite worms. For immunofluorescence/FISH experiment, more than 5 biological replicates (worms) were included for each group, and all shows consistent observation. For SILAC assay, 4 biological replicates were included. For single worm whole genome sequencing, more than 5 biological replicates were included.
Randomization	Randomization was not applied because the group allocation was guided based on the genotype of the respective mutant worms. Worms of a given genotype were nevertheless randomly selected from large strain populations for each experiment without any preconditioning.
Blinding	Blinding was not applied as the experiments were carried out under highly standardized and predefined conditions such that an investigator-induced bias can be excluded.

Reporting for specific materials, systems and methods

We require information from authors about some types of materials, experimental systems and methods used in many studies. Here, indicate whether each material, system or method listed is relevant to your study. If you are not sure if a list item applies to your research, read the appropriate section before selecting a response.

Materials & experimental systems

n/a	Involved in the study	
☐	☒	Antibodies
☒	☐	Eukaryotic cell lines
☒	☐	Palaeontology and archaeology
☐	☒	Animals and other organisms
☒	☐	Human research participants
☒	☐	Clinical data
☒	☐	Dual use research of concern

Methods

n/a	Involved in the study	
☒	☐	ChIP-seq
☒	☐	Flow cytometry
☒	☐	MRI-based neuroimaging

Antibodies

Antibodies used

Primary antibodies used for immunofluorescence staining are:
Rabbit polyclonal anti-phospho-RNA Polymerase II (Ser2) antibody (Thermo Fisher/Invitrogen,Cat. No.: A300-654A, dilution 1:500 in PBT);
Mouse monoclonal (mAbcam 1220) anti-H3K9me2 antibody (Abcam, ab1220, dilution 1:100 in PBT);
Rabbit polyclonal anti-HIM-8 (Novus Biologicals, Cat. No. 41980002, dilution 1:100 in PBT);
Rabbit anti-RAD-51 (N-terminal 103 amino acids) antibody is a homemade antibody and is a kind gift from Anton Gartner's lab (dilution 1:2000 in PBT).
Secondary antibodies used are:
AlexaFluor 488 donkey anti-mouse IgG (Thermo Fisher/Invitrogen, Cat. No.: A21202, dilution 1:500 in PBT) ;
AlexaFluor 594 donkey anti-rabbit IgG (Thermo Fisher/Invitrogen, Cat. No.: A21207, dilution 1:500 in PBT).

Validation

Rabbit polyclonal anti-phospho-RNA Polymerase II (Ser2) antibody (https://www.thermofisher.com/antibody/product/Phospho-RNA-Polymerase-II-Ser2-Antibody-Polyclonal/A300-654A)
Mouse monoclonal (clone mAbcam 1220) anti-H3K9me2 antibody (https://www.abcam.com/histone-h3-di-methyl-k9-antibody-mabcam-1220-chip-grade-ab1220.html)
Rabbit polyclonal anti-HIM-8 (https://www.novusbio.com/products/him-8-antibody_41980002)
Rabbit anti-RAD-51 antibody (https://link.springer.com/article/10.1007/s00412-003-0237-5)
Anti-mouse IgG (https://www.thermofisher.com/antibody/product/Donkey-anti-Mouse-IgG-H-L-Highly-Cross-Adsorbed-Secondary-Antibody-Polyclonal/A-21202)
Anti-rabbit IgG (https://www.thermofisher.com/antibody/product/Donkey-anti-Rabbit-IgG-H-L-Highly-Cross-Adsorbed-Secondary-Antibody-Polyclonal/A-21207)

Animals and other organisms

Policy information about studies involving animals; ARRIVE guidelines recommended for reporting animal research

Laboratory animals

Caenorhabditis elegans, hermaphrodites, age is day-1 adult. Strains used in this study are: N2 (Bristol; wildtype), CB4108 fog-2(q71), BC784 spe-8(hc50), RB1067 his-24(ok1024), MT13971 hpl-1(n4317), DW102 brc-1(tm1145), FX1524 cku-70(tm1524), FX2026 polq-1(tm2026), BJS1017 his-24(ok1024); fog-2(q71), BJS1018 hpl-1(n4317); fog-2(q71), BJS1019 brc-1(tm1145); fog-2(q71), BJS1020 cku-70(tm1524); fog-2(q71), BJS1021 polq-1(tm2026); fog-2(q71).

Wild animals

The study did not involve wild animals.

Field-collected samples

The study did not involve samples collected in the field

Ethics oversight

No ethical approval was required because C. elegans is a non-vertebrate species.

Note that full information on the approval of the study protocol must also be provided in the manuscript.
